# Tailorable, Lightweight and Superelastic Liquid Metal Monoliths for Multifunctional Electromagnetic Interference Shielding

**DOI:** 10.1007/s40820-021-00766-5

**Published:** 2021-12-13

**Authors:** Yadong Xu, Zhiqiang Lin, Krishnamoorthy Rajavel, Tao Zhao, Pengli Zhu, Yougen Hu, Rong Sun, Ching-Ping Wong

**Affiliations:** 1 Shenzhen Institute of Advanced Electronic Materials, Shenzhen Institute of Advanced Technology, Chinese Academy of Sciences, Shenzhen, 518055 People’s Republic of China; 2 School of Materials Science and Engineering, Georgia Institute of Technology, Atlanta, GA 30332 USA

**Keywords:** Liquid metal, Elastic monolith, Confined thermal expansion, Electromagnetic interference shielding, Magnetic actuation

## Abstract

- A confined thermal expansion strategy to fabricate liquid metal (LM)-based monoliths with continuous LM network at ultra-low content.
- The results show a strong integration advantage of LM-based monoliths in density, mechanical strength, electromagnetic interference shielding effectiveness, and near field shielding effectiveness, as well as multi-functions such as magnetic actuation.

**Abstract:**

Liquid metal (LM) has become an emerging material paradigm in the electromagnetic interference shielding field owing to its excellent electrical conductivity. However, the processing of lightweight bulk LM composites with finite package without leakage is still a great challenge, due to high surface tension and pump-out issues of LM. Here, a novel confined thermal expansion strategy based on expandable microsphere (EM) is proposed to develop a new class of LM-based monoliths with 3D continuous conductive network. The EM/LM monolith (EM/LMm) presents outstanding performance of lightweight like metallic aerogel (0.104 g cm^−1^), high strength (3.43 MPa), super elasticity (90% strain), as well as excellent tailor ability and recyclability, rely on its unique gas-filled closed-cellular structure and refined LM network. Moreover, the assembled highly conducting EM/LMm exhibits a recorded shielding effectiveness (98.7 dB) over a broad frequency range of 8.2–40 GHz among reported LM-based composites at an ultra-low content of LM, and demonstrates excellent electromagnetic sealing capacity in practical electronics. The ternary EM/LM/Ni monoliths fabricated by the same approach could be promising universal design principles for multifunctional LM composites, and applicable in magnetic responsive actuator.
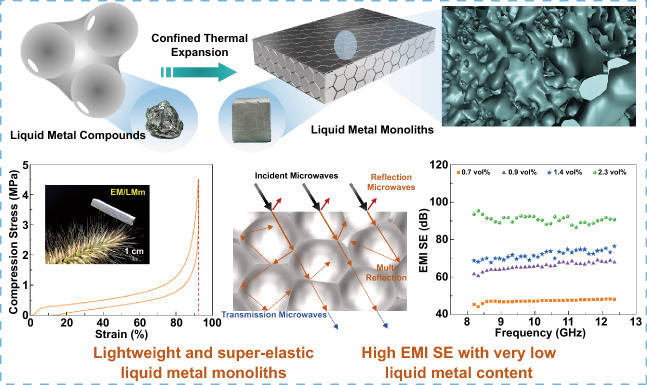

**Supplementary Information:**

The online version contains supplementary material available at 10.1007/s40820-021-00766-5.

## Introduction

Electromagnetic interference (EMI) caused by ubiquitous communication equipment is harmful to electronic products and the human body. Preparation of high-performance EMI shielding materials was widely studied as a means to effectively alleviate this dilemma [1–6], and the essence is to achieve attenuation of electromagnetic waves by constructing a complete Faraday cage through shielding material and matrix structure. Normally, interfaces and gaps are inevitably generated in this process, which are the main reasons for electromagnetic signal leakage and degradation of shielding effectiveness (SE). Therefore, the electromagnetic sealing at the gap is one of the most important factors that must be considered in actual applications, in addition to high conductivity and high intrinsic SE values.

Room temperature gallium-based liquid metal (LM) possesses fluidity and high electrical conductivity, which is different from the inflexible conductive network formed by traditional solid-phase conductive fillers [7–9]. Using the fluidity of LM to construct a conductive network with a dynamic response is expected to solve the leakage problem of electromagnetic sealing interfaces. However, the surface tension of LM causes its unique fluidity to be a double-edged sword in practical applications. LM network migrates and pumps out under external force, which severely limits its processibility and applicability. The main current processing strategies of LM-based composites are as follows: (1) top-down approaches [10–15], the bulk LM was divided into micro or nano-sized liquid drops, and (2) parallel approaches [16–21]. This strategy usually directly utilizes the bulk LM, such as injecting bulk LM into a precast channel, pouring bulk LM into porous foam, and blending bulk LM with metal powder. However, the current processing strategies require a large amount of LM to construct the functional network, and the weak self-sustainability further worsens the stability of LM, causing the leakage of LM and requiring an additional package.

Construction of LM-based conductive network at low filler contents along with highly self-standing, reliable, and reproducible flexible composite has great significance to extend practical applications of LM. Foaming is an effective method to construct conductive networks and reduce the content of fillers, including freeze-drying [22–24], supercritical carbon dioxide foaming [25, 26], chemical foaming [27], and sacrificial templates [3, 28], Unfortunately, the majority of traditional foaming process is not suitable to make LM-based polymer matrix foam structure due to migration and poor compatibility. Therefore, it is still a great challenge to develop an efficient and feasible processing method for the preparation of macroscopically self-standing, reliable LM composites.

## Experimental Section

### Preparation of EM/LM Compounds

The liquid metal (LM, eutectic indium gallium) with a melting point of 14.7 °C (Fig. S1) was fabricated by alloying 75 wt% gallium and 25 wt% indium (99.99%, DINGTAI metal materials, China). The above both are stirring at 60 °C until completely mixed. An expandable microsphere (EM, Expancel 031DU40) was purchased from AkzoNobel Co. Ltd, which is a hollow spherical polymer consisting of a thermoplastic closed shell and low boiling alkane trapped in the internal space. The EM/LM compounds were prepared through mechanical mixing of EM and LM following a specific volume ratio in the plastic container under the air environment.

### Preparation of EMm, EM/LMm and EM/LM/Ni Monoliths

A certain amount of the EM/LM compounds with different ratios were placed in a closed mold cavity with 2.5 × 1.5 × 0.5 cm^3^. After being heated at 95 °C for 1 h in a drying oven, the free-standing bulk EM/LMm without extra package were obtained. The EM/LM/Ni monoliths were also fabricated as similar to EM/LMm preparation excepting adding a certain of Ni power in the mixture of EM and LM. As a control, the pure EMm was also prepared by the same confined thermal expansion process.

Preparation of EM/LM/SI monoliths: Firstly, the EM/LM compounds were freely expanded at 95 °C for 1 h in an opened mold. Then, the liquid silicone (SI, A: B components with 1:1 weight ratio, CX-3425, TRANCY, China) was poured into the mold under a vacuum-assisted impregnation environment for 30 min to ensure full infiltration of liquid silicone into the gap of expanded EM/LM. Finally, the EM/LM/SI was heat cured at 60 °C for 2 h.

### Characterization

The morphologies and elemental analysis were viewed using a scanning electron microscope (SEM, Nova Nano 450, FEI, USA) and an energy-dispersive spectrometer (EDS, X-MaxN 80 T, Oxford, UK). All the samples were sputter-coated with gold before observation. The surface roughness of composites was tested by laser scanning confocal microscope (VK-X1000, KEYENCE, Japan). The contact angels of monoliths were measured by optical contact angel analyzers (OCA20, Dataphysics, German). A mechanical test machine (Japan Instrumentation System Co., LTD) with a force sensor of 500 N (JLC-M500N) was used to measure the compression stress–strain curves and the loading–unloading rate was set as 2 mm min^−1^. Electrical conductivity of EM/LM compounds was measured using Loresta-GP meter (MCP-T610, Mitsubishi Chemical, Japan) with a four-pin probe (PSP, MCP-TP06P). Electrical conductivity of EM/LMm was measured by the digital multimeter (Agilent 34401A) and calculated by the following equation:

1 $$\sigma = \frac{1}{\rho } = \frac{1}{R} \times \frac{l}{S} = \frac{1}{R} \times \frac{l}{t \times w}$$

where *ρ* (Ω m), *R* (Ω), *S* (m^2^), *l* (m), *t* (m), and *w* (m) are the resistivity, resistance, cross-sectional area, length, thickness, and width of the measured samples.

### EMI SE Performance Tests

The EMI SE properties of the EM/LMm were measured using a vector network analyzer (VNA, KEYSIGHT, N5227B, 10 MHz–67 GHz). The EMI SE measurement was performed using the wave-guide method. The EM/LMm cut into 22.86 × 10.16, 15.80 × 7.90, 10.67 × 4.32, and 7.12 × 3.56 mm^2^ were placed into a waveguide tube for measurements in the frequency range 8.2–12.4 (X-band), 12.4–18.0 (Ku-band), 17.6–26.7 (K-band), and 26.3–40.0 (Ka-band) GHz, respectively. The power coefficient of reflectivity (R), transmissivity (T), and absorptivity (A) can be obtained from the measured scattering parameters (*S*_11_, *S*_21_), and then the total EMI SE (SE_T_), microwave reflection (SE_R_), and microwave absorption (SE_A_) can be calculated as follows:

2 $$R = \left| {S_{11} } \right|^{2}$$

3 $$T = \left| {S_{21} } \right|^{2}$$

4 $$1 = A + R + T$$

5 $${\text{SE}}_{{\text{T}}} = - 10\log T$$

6 $${\text{SE}}_{{\text{R}}} = - 10\log \left( {1 - R} \right)$$

7 $${\text{SE}}_{{\text{A}}} = - 10\log \left( {\frac{T}{1 - R}} \right) = {\text{SE}}_{{\text{T}}} - {\text{SE}}_{{\text{R}}} - {\text{SE}}_{{\text{M}}}$$

### Near-field Shielding Effectiveness (NF-SE) Performance Tests

The NF-SE measurement system (Smart Scan-350/550 EMI API) is shown in Fig. 5a, which is composed of a printed circuit board (PCB) board, micro-strip antenna, metal frame, and shielding materials. A patterned micro-strip antenna was used to generate the signal fields. A scanning probe was employed to capture a near field signal leakage (magnetic field signal, H) from the covered shielding materials, and the scanning was performed using a precise positioning robotic arm. One end of the micro-strip antenna and the scanning probe was connected to the port 1 and port 2 of VNA, respectively. NF-SE extracted from 2-port *S*-parameters measurement of *S*_21_. Here, the shielding materials at a thickness of 4 mm were covered in the metal frame and fixed through bolts.

## Results and Discussion

**Fig. 1:**
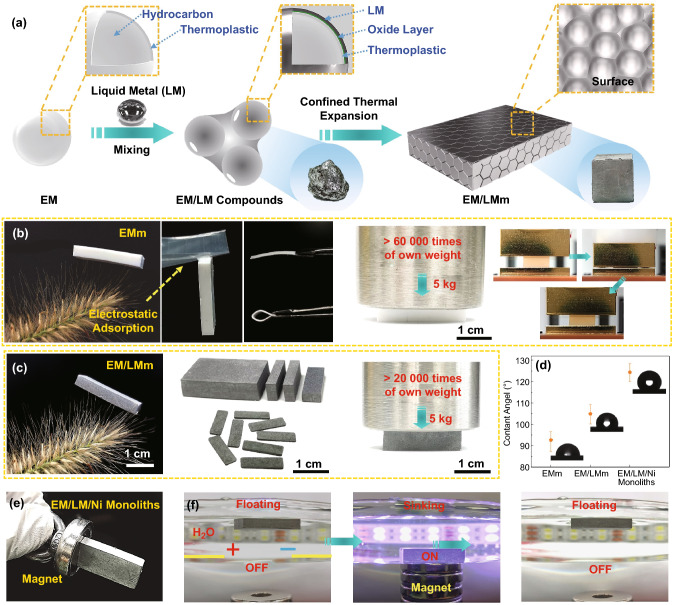
Schematic of EM/LMm preparation and macroscopic features demonstration. **a** Illustration of the fabrication process of EM/LMm and forming mechanism. **b**, **c** Optical photographs of pure EMm and EM/LMm show low density, super elastic, tailorability, and high load-carrying ability. **d** The contact angle of different monoliths. **e**, **f** The demonstration of EM/LM/Ni monoliths as underwater magnetic-controllable switch

Here, we propose a novel confined thermal expansion strategy to fabricate stable LM-based monoliths with 3D continuous conductive network at ultra-low LM contents, which are tailorable, lightweight, high strength, superelastic, highly electrically conductive, and outstanding EMI shielding performance, highlighting features imparted by expandable microspheres (EM) and LM. Figure 1a is the schematic fabrication process of EM/LM monoliths (EM/LMm) through a confined thermal expansion approach. The EM and LM were mixed by constant mechanical stir in the air atmosphere. In this process, the EM is firstly attached to on the surface of the LM by electrostatic adsorption (Fig. S2). Under mechanical stirring, the surface oxide layer of LM was continually ruptured and adhered to on the surface of EM (Fig. S3). It should be noted that this wetting behavior is dominated by the intimate contact between new oxide grown at the oxide fracture and EM [29]. The new oxide has a surface of lower effective roughness compared old oxide layer so that it is more prone to adhesion. Finally, EM are gradually dragged into the LM with the oxide, and then a new oxidation layer is formed on the LM surface. The EM powders are loosely connected with each other by continuous LM, forming a plasticine-like paste. For constructed refined LM networks, the EM/LM compounds were placed into a sealed mold at 95 °C above the glass-transition temperature of the EM shell for 1 h. During hydrocarbon gasification, the volume of EM increased drastically within the sphere structure from 5 ~ 15 to 10 ~ 60 μm (Fig. S4). And also, expansion of EM is favorable for selective distribution of LM between EM interstitial spaces while achieving self-sintering of EM shell under heat and pressure within the mold space. For comparison, pure EM monoliths (EMm) (Fig. 1b), and EM/LMm (Fig. 1c) were fabricated by confined thermal expansion method and possessed lightweight with the density of 0.034 and 0.104 g cm^−3^, respectively. The assembled monoliths can be easily supported by the bristlegrass without visible bending and are also tailored into thinner pieces with excellent flexibility and bendability. It is interesting to observe that the pure EMm can adhere to polyethylene film via the electrostatic adsorption, however, EM/LMm does not exit electrostatic adsorption due to the presence of conducting liquid metal. Moreover, both the EMm and EM/LMm exhibit remarkable mechanical strength and outstanding elasticity, which can bear heavy objects over 60,000 and 20,000 times of their own weights, respectively, and also nearly recover to its initial state after compressed more than 90% of strain (Movie S1). More importantly, the EMm and EM/LMm can be easily scaled up via the confined thermal expansion method and reprocessed to various shapes such as disk, triangle, and cube (Fig. S5 and Movie S2). In addition to that, the measured contact angle of EMm is 92.6° and increases to 104.9° and 124.4° for EM/LMm and EM/LM/Ni monoliths, respectively, indicating that a promising candidate is a lightweight functional underwater component (Fig. 1d). The multifunctional EM/LM/Ni monoliths were also successfully fabricated by the same confined thermal expansion method with the filling of nickel particles into liquid metal exhibited unique magnetism (Fig. 1e). The ternary EM/LM/Ni monoliths can be used as a promising magnetic-floating switch to control OFF and ON state for the designed circuit and LEDs through an external magnetic field (Fig. 1f and Movie S3), which demonstrates a great potential for future magnetic responsive actuators.

**Fig. 2:**
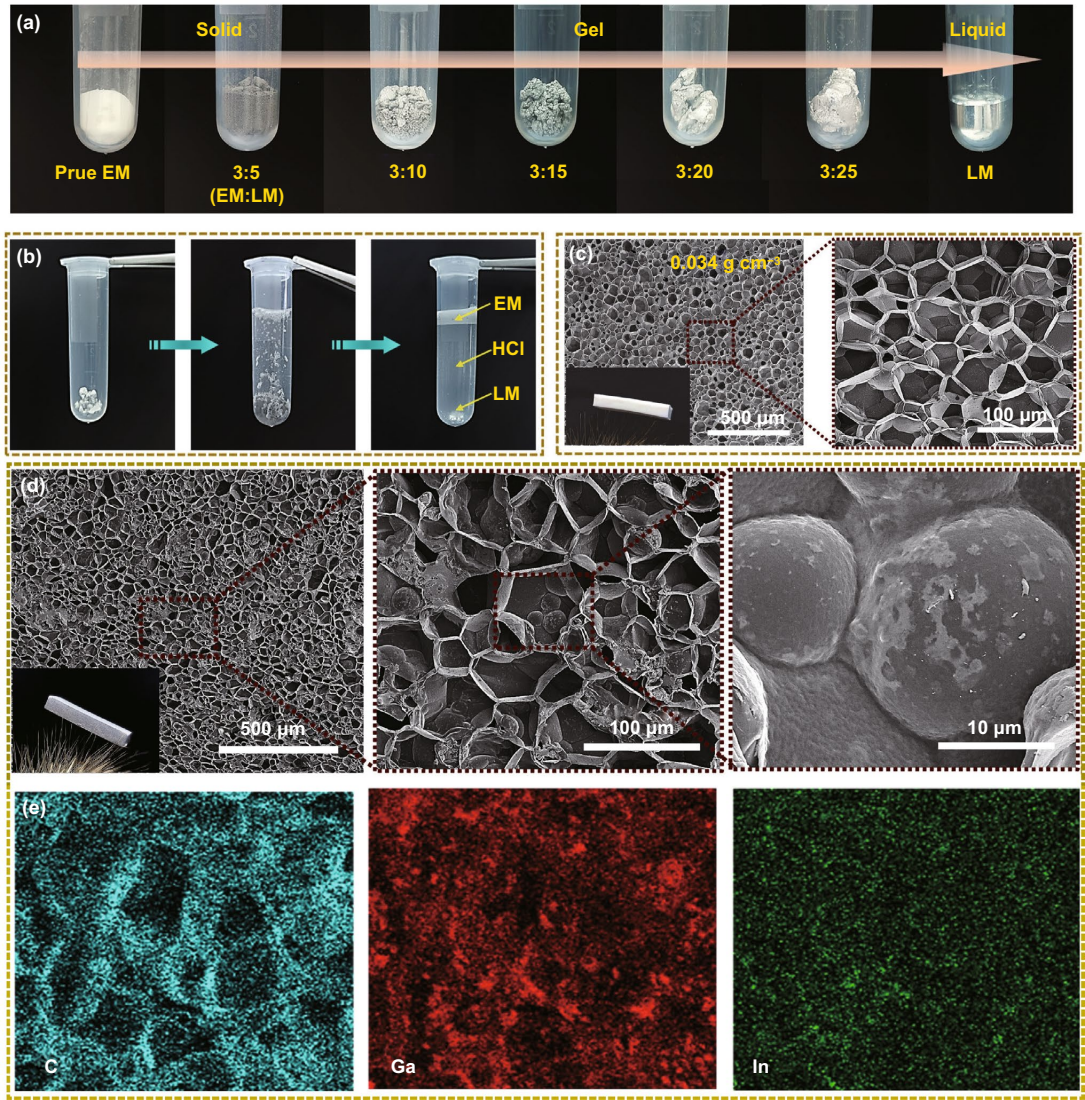
Structural characterization of EM/LM compounds, EMm and EM/LMm. **a** Optical photographs of EM/LM compounds at various ratios, beginning as EM and transitioning from a solid-like to a liquid-like material as more LM added. **b** Demonstration of recyclability of EM/LM compounds, automatic separation of two components depends on the density difference in dilute HCl solution. **c** Cross-sectional SEM images of EMm at the density of 0.034 g cm^−3^. **d** Cross-sectional SEM images of EM/LMm at the density of 0.126 g cm^−3^. **e** EDS mapping of cross-sectional EM/LMm and its uniform distribution of Ga and In elements

High surface tension and leakage of LM affect its operability. At present, the most common method is to use the oxide layer composed of Ga_2_O_3_ formed on the surface of LM to improve wettability and then assembled as a composite with different polymer matrices [30–33]. However, the passivated oxide layers need to be reactivated to maintain the characteristics of LM itself [28, 34–36]. The ideal approach is to improve the processability of LM and nullify the influence of the oxide layer during the assembly of a continuous electrical conducting network in fabricated LM composites. Hence, the EM with thermal self-expanding property was introduced into LM to achieve the refined construction of a highly conductive LM network. Through adequate mechanical mixing, the oxide layer was continuously generated to realize the coating of EM by LM. Figure 2a shows that the EM/LM compounds with different rheological properties, beginning as a pure solid EM and transition into liquid phase as more LM was added. In particular, gel-like EM/LM compounds with plasticine features can be observed by adjusting the mixing ratio due to the cohesion brought by the LM (Fig. S6). The SEM images of EM/LM compounds (Fig. S7) show uniform distribution without particle agglomeration and sedimentation relying on the low density of EM. The volume repulsion of EM effectively reduces the density of EM/LM compounds (varying from 1.62 to 0.63 g cm^−3^) while ensuring the high conductivity up to 1.06 × 10^6^ S m^−1^ (Fig. S7). In consideration of the rising requirement of green processing and high cost of liquid metal, the recycling of LM is extremely important. As shown in Fig. 2b, when EM/LM compounds were immersed in 1.0 M dilute hydrochloric acid solution at room temperature, the surface gallium oxide was dissolved by HCl leads to gravimetric sedimentation of lightweight EM and heavy LM, which depicts that an efficient and eco-friendly recycling strategy of LM.

In order to obtain free-standing bulk LM-based composites, the confined thermal expansion process was employed by heating the mixture of LM and EM in a homemade closed mold with designed size. The cross-sectional SEM images of prepared pure brittle fracture EMm show densely packed closed-cellular structure (Fig. S8). The pure EM spheres strongly squeeze and adhere each other, leaving sintering traces on the surface. In particular, unlike the thermal expansion of EM in open space, the confined thermal expansion achieved the self-sintering effect between EM’s. It should be noted that the self-sintering process determined by the combination of confined mold and cell pressure, results from the internal gas expansion and shell softening at a given temperature field. The cross-sectional SEM images of EMm obtained by razor cutting show a typical cellular structure (Fig. 2c). The density of EMm can be controlled by adjusting the amount of EM added into the mold. Due to the confined space in the closed mold, the denser EMm with increasing density from 0.034, 0.056 to 0.078 g cm^−3^ formed, which has a smaller average cell pore size from about 50, 30 to 20 μm, respectively. (Fig. S9).

After introducing the LM with fluidity, the expansion process of EM/LM compounds in open space exhibits high synchronization. The EM/LM compounds remark negligible change in mass and phase separation during open thermal expansion, yet obviously increasing in volume (Fig. S10 and Movie S4). The higher interfacial wettability between the LM and EM leads to the relative high viscosity of LM thus, LM were uniformly distributed among EM spheres forming the continuous conductive phase. More importantly, the passivation oxide layer in LM is broken during the thermal expansion process resultant the formation of continuous interconnected LM conductive paths, which is significantly different from many traditional methods to build the LM conductive network by extra processes such as compress [28, 36, 37], stretch [20, 38], and freeze [34, 39]. By comparison, EM/LMm were fabricated by confined thermal expansion strategy. The cross-sectional SEM images of the EM/LMm still present a close-packed regular cellular structure (Fig. 2d), which is evidence that the LM does not hinder the sintering of EM. The corresponding EDS diagram further confirms the uniform distribution of LM in EM/LMm (Fig. 2e). Cross-sectional SEM images of the EM/LMm at different locations show that the similar and sufficient LM distribution from bottom to top, which reveals that the LM-driven by the expansion of EM and high efficacy making monolith structures (Fig. S11).

**Fig. 3:**
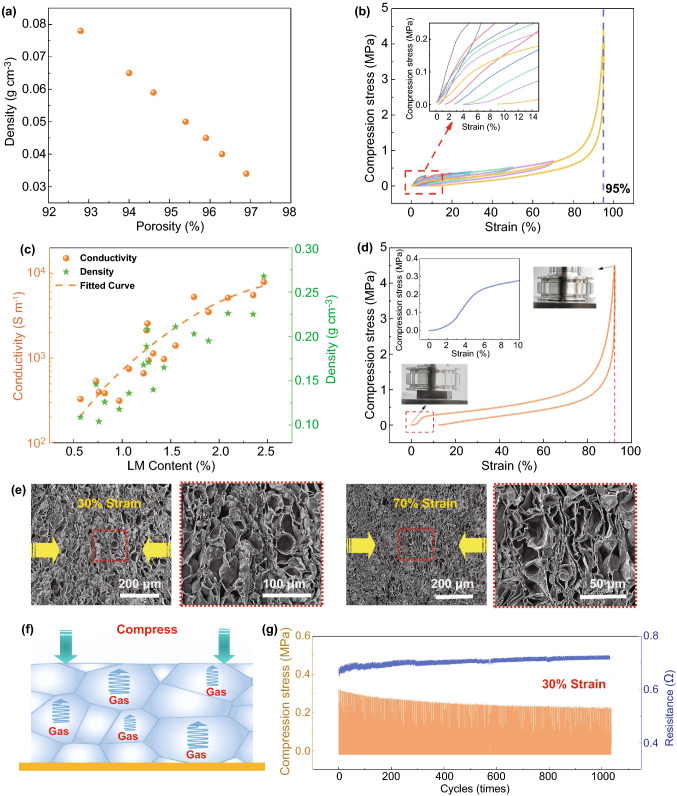
Density, electrical and mechanical properties of the EMm and EM/LMm. **a** The density of EMm corresponding to porosity. **b** Compression stress–strain curves of EMm (density: 0.054 g cm^−3^) under different strains. **c** The conductivity and density of EM/LMm depend on the LM content. **d** Compressive stress–strain curve of EM/LMm (density: 0.126 g cm^−3^) at 90% strain. **e** SEM images show deformation of cell structure at the compressive strain of 30% and 70%. **f** Schematic illustration dynamic elastic behaviors of the EM/LMm upon mechanical compression-releasing. **g** Mechanical–electrical properties of the EM/LMm under loading–unloading cycles

The density of the EMm can be tuned from 0.034 to 0.078 g cm^−3^, and its corresponding porosity varied between 96.9 and 92.8% (Fig. 3a). The compression performance test of EMm shows that both high compressive strength of 4.49 MPa at 95% strain and superior resilience of more than 90% recovery after releasing the pressure (Fig. 3b). It is worth noting that our fabricated lightweight EMm with both high compressive strength and high elasticity, has rarely been reported in the previous research (Table S1). Nevertheless, the EMm with an unique gas-filled closed-cellular structure demonstrated an efficient stress dissipation process, and also spanned from each cellular unit to internal gas. As the volume rapidly decreased during compressive cycles, the cellular unit pressure increases gradually, resulting in an increase in the stress modulus and resilience. In addition to that, by the introduction of LM in EMm further endows conductive properties to the composites. Figure 3c shows the change in conductivity of EM/LMm for different LM contents. At 2.46 vol% of LM contents, the electrical conductivity of the EM/LMm has reached about 7891 S m^−1^. The traditional solid-phase filler materials form a conductive network through overlapping each other [22, 40–42]. Beneficially, LM form the perfect continuous interconnected network with affordable lower contact resistance due to intact intrinsic conductivity of LM, which was not deteriorated during the confined thermal expansion process as like traditional LM composite fabrications. In addition, the density of EM/LMm increases with the LM content. Fortunately, the value is still in a low range of about 0.104–0.268 g cm^−3^ due to low LM filling content originating from our unique and novel processing method, which is the lowest value of LM-based conductive composites in previous reports to the best of our knowledge [19, 43]. It can be seen from the above structural characterization (Fig. 2) that the EM/LMm maintains a nearly identical cellular structure with EMm. The EM/LMm still shows remarkable compressive strength of 3.43 MPa at 90% strain and a high recovery ratio of 88.5% even exceeding 90% compressive strain (Fig. 3d). The compression strength of EM/LMm increases with decreasing density (Fig. S12), which can be attributed to the specific forming method of EM/LMm.

Here, the specific compression strength (SCS, Nm kg^−1^) defined as strength (N m^−2^) divided by material density (kg m^−3^) is used to describe the lightweight and high strength performance of materials. Compared to other compressible elastic polymer-based foam, the EMm shows a higher SCS value about 10,400 Nm kg^−1^ (Table S1) consistent with the ultra-high load-carrying ability (over 60,000 times of its own weight) as shown in Fig. 1b. The density of the EM/LMm was increased upon the addition of LM, however, the SCS still reaches 2,711 Nm kg^−1^ and the EM/LMm maintains extremely high load-carrying ability and resilience. The critical role of such gas-filled closed-cellular structures related to high elasticity, and strength during compression cycles was further explored by recording cross-sectional SEM morphology of EM/LMm in different compression strains. As supported from Fig. 3e, the pore walls of the monolith are oriented opposite to the direction of applied strain at both lower (30%) and higher strain (70%) to compromise the strain load. In general, the lightweight and high strength foam structure, this kind of deformation during compressive strain was often irreversible [44–48]. However, the EM/LMm can recover quickly when the stress was released, which results from the special gas filling structure and also assembly of a closed-cell structure of EM/LMm. In addition to the pore wall of the EM/LMm, the internal gas can also be compressed to disperse the stress further enhancing the rebound power during the recovery process (Fig. 3f). Thanks to the synergy of thousands of closed-cellular structures, the EM/LMm exhibits high compression strength and resilience. After repeated compression-release tests over 1000 times, the EM/LMm shows a little stress reduction, mainly due to the stress relaxation of the polymer pore wall during the repeated compression process (Fig. 3g). Moreover, the electrical conductivity of the EM/LMm shows high stability during the cyclic test with a resistance tolerance limit of about ± 0.14 Ω. Besides, the tensile performance results of EMm show satisfactory tensile strength (0.79 MPa) and elongation at break (172.2%), which was mainly due to the in-situ sintering of the microsphere wall under high pressure (Fig. S13). At the same time, the introduction of liquid metal weakened the sintering effect between the cell walls to a certain extent, resulting in a decrease in tensile strength and elongation at the break of EM/LMm. More importantly, with advances in controlling LM formation, our adopted thermal expansion strategy of making EM/LMm opens up more processing possibilities for composites with other features. The EM/LM compounds were expanded freely in advance to realize the construction of the LM network. And then the liquid silicone (SI) was poured into the compounds and heat cured in a vacuum to obtain the conductive EM/LM/SI monoliths. The cyclic compression performance curve of EM/LM/SI monoliths shows a high degree of mechanical–electrical stability (Fig. S14). After 100 cycles of compression testing, the compressive strength of EM/LM/SI monoliths decays somewhat, mainly due to the weak bonding force between EM (Fig. S15).

**Fig. 4:**
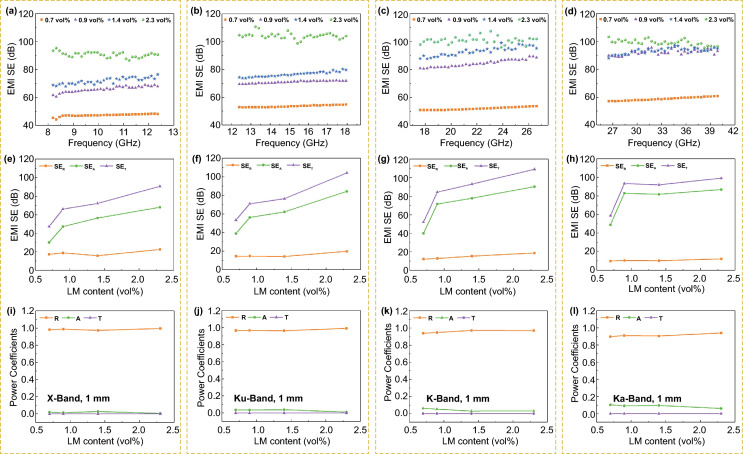
The EMI SE performance and shielding mechanism of EM/LMm with various LM contents at a thickness of 1 mm in 8.2–40 GHz. **a**–**d** The EMI SE of EM/LMm with various LM contents in different frequency ranges. **e**–**h** Comparison of EMI shielding mechanism of EM/LMm with various LM contents (the average SE_R_, SE_A_, and SE_T_ in different frequency ranges). **i**–**l** Average power coefficients of R, A, and T of EM/LMm at different frequency ranges

The EM/LMm with excellent conductivity offer outstanding EMI shielding performance. The EMI shielding effectiveness (SE) of EM/LMm was measured using a vector network analyzer (VNA) in a broadband frequency range of 8.2–40 GHz, including X-band, Ku-band, K-band, and Ka-band (Fig. 4). Compared with the negligible EMI SE (0.048 dB in X-band) of EMm (Fig. S16), The EMI SE of EM/LMm can be optimized by adjusting the LM content, and over 40 dB values were obtained at only 0.9 vol% LM with the thickness of 1 mm. The average EMI SE of EM/LMm sample with 2.3 vol% of LM in X-band reaches up to 90.6 dB, and the EMI SE increases over 100 dB with the increase of frequency (Fig. 4a–d). The high EMI shielding performance of EM/LMm mainly originates from its dense continuous LM network (Fig. S17). When an electromagnetic wave encounters the LM skeleton, the scattering of the incident wave occurs and dramatically reduces the intensity of the microwaves. The connected LM skeleton structure can attenuate the incident electromagnetic waves by scattering, reflecting, and absorbing [49, 50]. As we know, the high-frequency electromagnetic waves will attenuate faster in the medium due to their shorter wavelength results in a closer distance between the troughs and crests. The greater difference in the electric field near a certain point of the medium causes the increasing current, therefore, the more electromagnetic waves energy is attenuated in the medium upon increasing frequency [6, 51]. EMI shielding mechanism analysis can also verify this trend. As shown in Fig. 4e–h, the total EMI SE (SE_T_) generally includes SE_R_ (reflection loss), SE_A_ (absorption loss), and SE_M_ (multi reflection loss), which depend on the wave impedance, mobile charge carriers, magnetic dipole, and interfaces of shielding material, respectively [52]. In a certain frequency range, the SE_A_ has a significant increase with the increase of LM content in EM/LMm, whereas the change in SE_R_ is not obvious. Besides, a higher SE_A_ and lower SE_R_ of EM/LMm was observed with the increase of frequency, which means that the EM/LMm have a stronger absorption ability and lower reflection of electromagnetic waves. However, it does not mean that the EM/LMm is an absorption-dominated shielding material, which can be further evaluated by analyzing the reflectivity (R), absorptivity (A), and transmissivity (T) co-efficient of EM/LMm. Figure 4i–l exhibits more than 90% reflectivity of EM/LMm in all measured frequency ranges, revealing that the shielding mechanism of EM/LMm to electromagnetic wave is still dominated by reflection. According to the plane wave models of the EMI shielding (more details are displayed in EMI shielding mechanism in Supporting Information), the SE_R_ is positively correlated with the difference of wave impedance between shielding materials and free space. For plane waves, the impedance of the free space wave is constant (377 Ω), and the impedance of the shielding material increases as the frequency increases. Hence, the SE_R_ decreases, and the SE_A_ increases, resulting in the improvement of SE_T_ with the increase of frequency. Besides, increasing the thickness of EM/LMm can greatly improve the EMI SE values of 72.2–86.8 dB at a thickness of 1–2 mm at 1.4 vol% of LM in X-band, respectively (Fig. S18). The influence of LM on the thermal conductivity of the EM/LMm was further explored (Fig. S19). Pure EMm exhibits extremely low thermal conductivity (0.02 W m^−1^ K^−1^) and high interface thermal impedance (1230.9 cm^2^ K W), due to the porous structure and low thermal conductive polymeric walls. The heat transfer inside the material is hindered. After the introduction of LM, the heat conduction path and efficiency inside the EM/LMm are significantly improved. The thermal conductivity of EM/LMm increases to 0.12 W m^−1^ K^−1^, and the interface thermal impedance reduces to 171.2 cm^2^ K W.

**Fig. 5:**
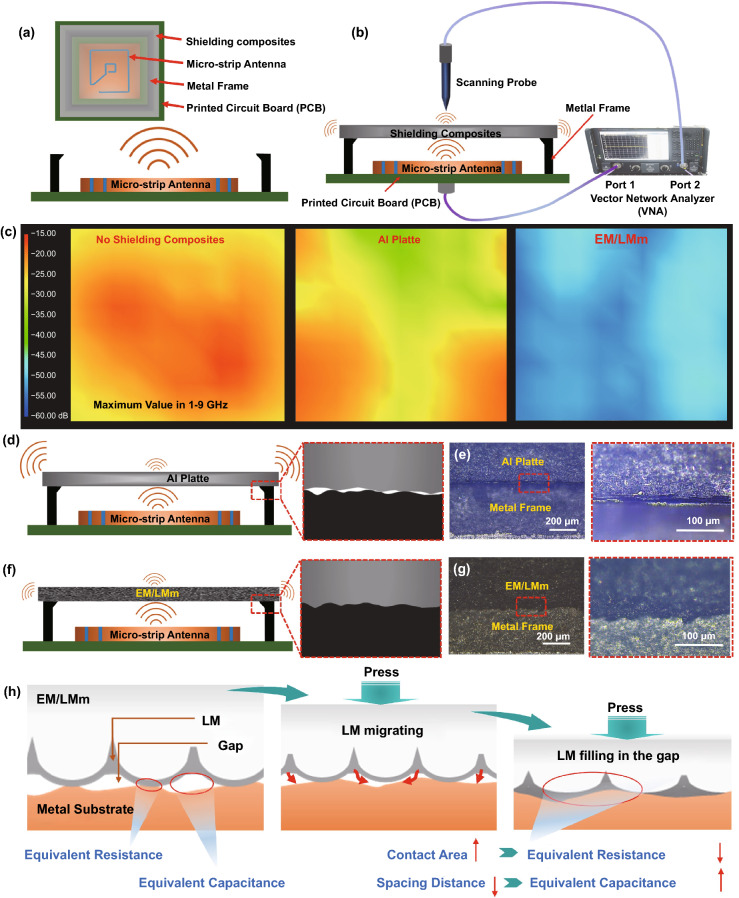
NF-SE performance of EM/LMm and the comparison of EM/LMm with other LM and shielding materials. **a**, **b** Schematic depicting the NF-SE testing principle and system. This testing model is mainly to measure the near field EMI SE performance of shielding materials by testing the relative electric and magnetic signals. **c** NF-SE mapping images of bare antenna, shielding with Al plate and shielding with EM/LMm (maximum value in every point among 1–9 GHz). **d**, **e** Schematic of shielding model with Al plate, and the optical images of the interface between the metal frame and Al plate. The holes were caused by the hard surface of the metal frame and Al plate. **f**, **g** Schematic of shielding model with EM/LMm. The optical images between the metal frame and EM/LMm show a tight interface. h The schematic diagram of interface electromagnetic self-sealing mechanism of EM/LMm

In practice, EMI shielding materials are required in conjunction with other components to form a complete enclosed Faraday cage for attenuating electromagnetic waves. One of the main causes of reduced electromagnetic sealing is the gap between the EMI shielding material and the component. Unfortunately, researchers paid more attention to the SE of shielding materials themselves in the past, while ignoring this problem faced by the shielding materials in actual application conditions. Here, we conducted a comparative study on shielding materials with different characteristics through the NF-SE test system (Fig. S20a) and found that the sealing of the shielding materials has a more important impact on the comprehensive EMI SE. As shown in Fig. 5a, the NF-SE test model is based on the micro-strip antenna launch system embedded in a printed circuit board (PCB) with a rectangular metal frame. The tested sample was placed and fully covered on the top of the metal frame to form a Faraday cage to isolate the electromagnetic wave signal (Fig. 5b). VNA provide a signal source for the micro-strip antenna connected to port 1, and the NF-SE was measured by a scanning probe with an automatic manipulator connected to the port 2. The upper radiation magnetic field intensity (magnetic field signal is more likely to leak than electric field signal) of shielding material is scanned and measured to comprehensively evaluate the EMI SE. Initially, we tested the average magnetic field intensity of different shielding materials in the frequency range of 1–9 GHz. The NF-SE mapping image demonstrates natural attenuation characteristics of electromagnetic waves, even without shielding materials (Fig. S20b). The upper average magnetic field intensity of micro-strip antenna explains characteristics of strong in the central area and weak in the surrounding area. Here, the NF-SE without shielding material is set as the baseline. Both of the aluminum (Al) plate and EM/LMm exhibit excellent average NF-SE in the frequency range of 1–9 GHz (Fig. S20c, d). However, the magnetic field intensity above the Al plate is majorly concentrated in the surrounding area, indicating that the leakage of magnetic field signal around the shielding material. The EM/LMm shows a uniform and weak magnetic field intensity distribution explicating excellent NF-SE and electromagnetic sealing properties. In order to further illustrate the importance of electromagnetic sealing, we make a surface scan and take the worst NF-SE values in the frequency range of 1–9 GHz in each area above the shielding materials. The Al plate shows serious electromagnetic wave leakage at a specific frequency, causing its comprehensive NF-SE to drop sharply with frequency changes although its average NF-SE is high (Fig. 5c, and S20e, f). Comparing EM/LMm, the comprehensive NF-SE does not fluctuate greatly with frequency. The observed results indicate the electrical conductivity of material not majorly contributed to the comprehensive performance in NF-SE. On the meta-microscale, there are actually a lot of gaps between the shielding material and the existing metal frame, which can be equivalent to a parallel circuit of multiple resistors and capacitors for analysis. Reducing resistance and increasing capacitance are both effective measures to improve the NF-SE. Figure 5d, e shows a large number of unfilled air gaps at the contact interface due to the existing surface roughness of metal frame (Fig. S21a-c) and mechanical rigidness of Al plate, resulting in increase in resistance and a decrease in capacitance. As for EM/LMm (Fig. 5f, g), the compressible properties and uneven surface at the microscopic scale greatly increase the contact area between them and reduce the distance of interface gaps. At the same time, a small amount of LM on the surface can further diffuse and bridge the interface gap under the anchoring pressure, which can be equivalent to an increase in capacitance and a decrease in resistance, respectively (Figs. 5h, and S21d-j). Therefore, the EM/LMm shows outstanding comprehensive performance in NF-SE. Further comparing the NF-SE of commercial plating nickel foam with Al plate and EM/LMm in different expressions (Fig. S20e, f), it can be seen that the EM/LMm has unique advantages in the stability of shielding and the independence of frequency. From experimental results of our fabricated EM/LMm exhibits outstanding advantages in multi-dimensional when compared to currently available LM-based composites and EMI shielding composites (Fig. S22, Tables S2 and S3).

## Conclusions

This is the first report of lightweight liquid metal-based bulk composites without leakage to the best of our knowledge. Although liquid metal composites have been very recently demonstrated in stretchable conductors, printed electronics, EMI shielding materials, etc., the leakage, high filling content, and high density have not been solved yet. Our strategy of confined thermal expansion of processing liquid metal provided a new universal way to design high-performance liquid metal-based composites with tailorability, low density, superelastic, high load-carrying capacity, and high electrical conductivity. The as-prepared EM/LMm realized the structural adjustment of liquid metal architectures from the microcosmic fluidity to the macroscopic stability and shows great advantages in electromagnetic shielding and sealing. We also demonstrate the multifunctional liquid metal-based composites achieved by the same strategy, which exhibit fascinating prospects in magnetic actuation, intelligent response, and smart sensors, etc. All in all, this work opens up a clear path that liquid metal can be handled to “solid metal” without the assistance of external packaging, and provides a prototype of multifunctional liquid metal composites, which may be extended to other functional viscous liquids.

## Supplementary Information

Below is the link to the electronic supplementary material.

Supplementary file1 (MP4 1177 kb)

Supplementary file2 (MP4 14268 kb)

Supplementary file3 (MP4 4316 kb)

Supplementary file4 (MP4 17364 kb)

Supplementary file5 (PDF 2645 kb)
