# Supplementary material for: Tailorable, Lightweight and Superelastic Liquid Metal Monoliths for Multifunctional Electromagnetic Interference Shielding
Source: Nanomicro Lett. 2021 Dec 13;14:29. doi: 10.1007/s40820-021-00766-5 (PMC8669089; doi:10.1007/s40820-021-00766-5)
Supplement: Supplementary file 5 — Supplementary file5 (PDF 2645 kb) [file 40820_2021_766_MOESM5_ESM.pdf]

Supporting Information for

Tailorable, Lightweight and Superelastic Liquid Metal Monoliths for Multifunctional Electromagnetic Interference Shielding

Yadong Xu[1], Zhiqiang Lin[1], Krishnamoorthy Rajavel[1], Tao Zhao[1], Pengli Zhu[1], Yougen Hu[1,]*, Rong Sun[1,]*, Ching-Ping Wong[2]

[1]Shenzhen Institute of Advanced Electronic Materials, Shenzhen Institute of Advanced Technology, Chinese Academy of Sciences, Shenzhen, 518055, P. R. China

[2]School of Materials Science and Engineering, Georgia Institute of Technology, Atlanta, GA, 30332, USA

*Corresponding authors. E-mail: yg.hu@siat.ac.cn (Yougen Hu), rong.sun@siat.ac.cn (Rong Sun)

S1 Supplementary Note

EMI shielding mechanism

According to Schelkunoff theory, the shielding mechanism of shielding materials can be divided into three parts：

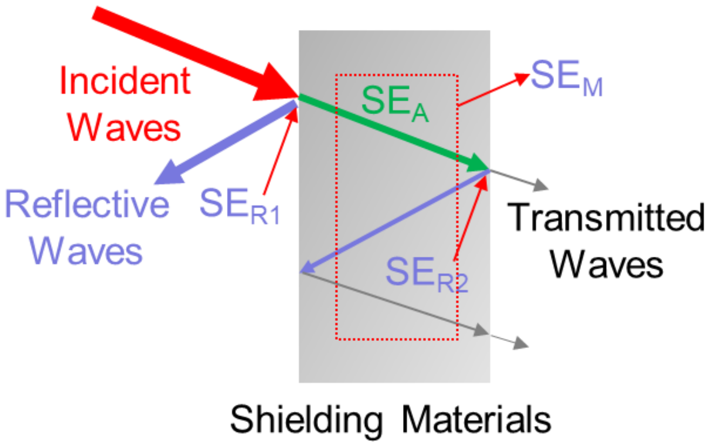

$$\text{EMI SE} = SE_A + SE_{R1} + SE_{R2} + SE_M = SE_A + SE_R + SE_M \quad \text{(S1)}$$

The SE_A is caused by the heat loss, which result from the electromagnetic coupling when electromagnetic waves passing through the shielding material. Here, the SE_A is expressed by the relative conductivity and permeability of shielding material:

$$SE_A = 0.131t\sqrt{f\mu_r\sigma_r} \qquad \text{(S2)}$$

Form formula above, the SE_A is positively correlated with frequency, electromagnetic parameters and thickness of shielding materials.

SE_R is caused by impedance discontinuity (impedance mismatch) at the surface of the shielding materials. The wave impedance of free space (Z_1) is much larger than that of shielding materials (Z_2). The SE_R is expressed as follow:

$$SE_R \approx 20lg\left[\frac{|Z_1|}{|4Z_2|}\right] \qquad \text{(S3)}$$

$$Z_2 = 3.68 \times 10^{-7}\sqrt{f\mu_r/\sigma_r} \qquad \text{(S4)}$$

When the electromagnetic wave is far field waves, the Z_1 is 377 Ω. The formula further evolves as follow:

$$SE_R = 168 + 10\,lg\frac{\sigma_r}{f\mu_r} \qquad \text{(S5)}$$

The SE_R is mainly depending on the wave impendence of shielding materials (Z_2).

The SE_M is multiple reflection correction factor, which refers to the electromagnetic waves being multiple reflected between the internal interfaces of the shielding material. After being reflected on the second interface of the shielding materials, the electromagnetic waves will continue to travel, and be reflected by the first interface again. When electromagnetic waves reach the second interface, part of them will penetrate the second interface and leak into the space. This part is extra leakage and should be considered in the calculation of EMI SE. Here, the SE_M is a negative value, and its role is to reduce the shielding effectiveness. The SE_M in here is related to the built model, and only suitable for theoretical calculations of EMI SE, which is different from the multiple reflection loss achieved by using multiple interfaces in design the shielding material.

$$SE_M = 20lg\left(1 - e^{-2t/\delta}\right) \qquad \text{(S6)}$$

$$\delta = 1/\sqrt{\pi f\mu\sigma} \qquad \text{(S7)}$$

$$\mu = \mu_0\mu_r \qquad \text{(S8)}$$

Where t is the thickness of the shielding materials (mm), f is the electromagnetic waves frequency. μ_r is the relative permeability of the shielding materials, $\mu_0=4\pi\times10^{-7}$ H.m^{-1}, σ_r is the conductivity of the shielding materials relative to copper. σ is the conductivity (S/m), δ is the skin depth.

S2 Supplementary Figures and Tables

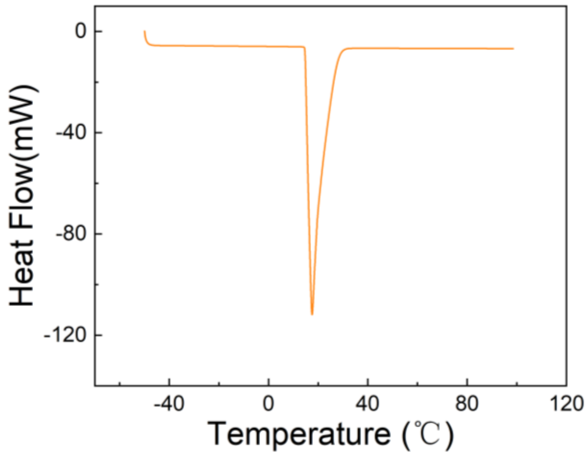

Fig. S1 The differential scanning calorimetry (DSC) curve of LM

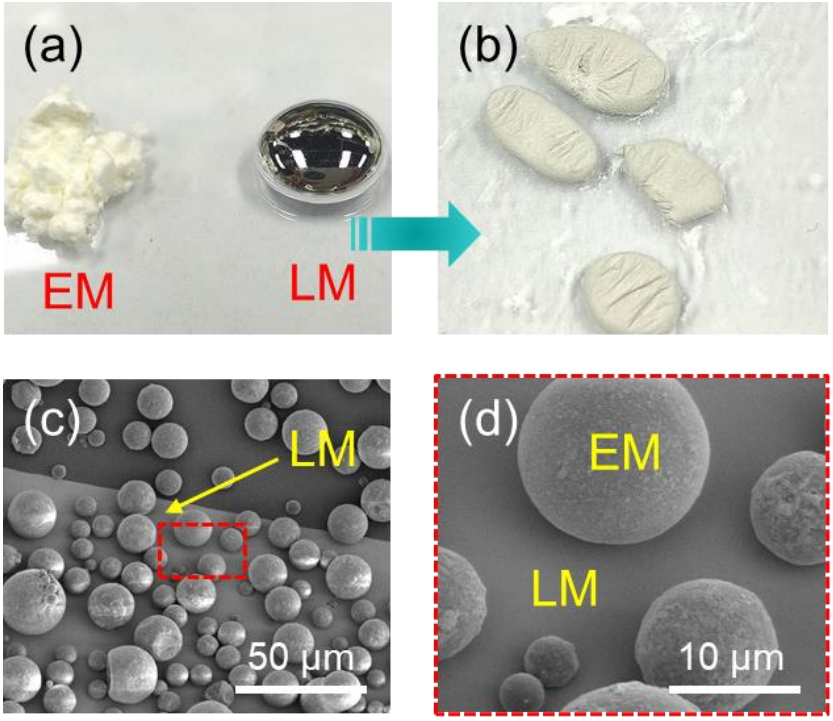

Fig. S2 Mixing process of EM and LM. (**a, b**) Optical images of EM and LM before and after mixing. (**c, d**) SEM images of EM adhered to LM surface

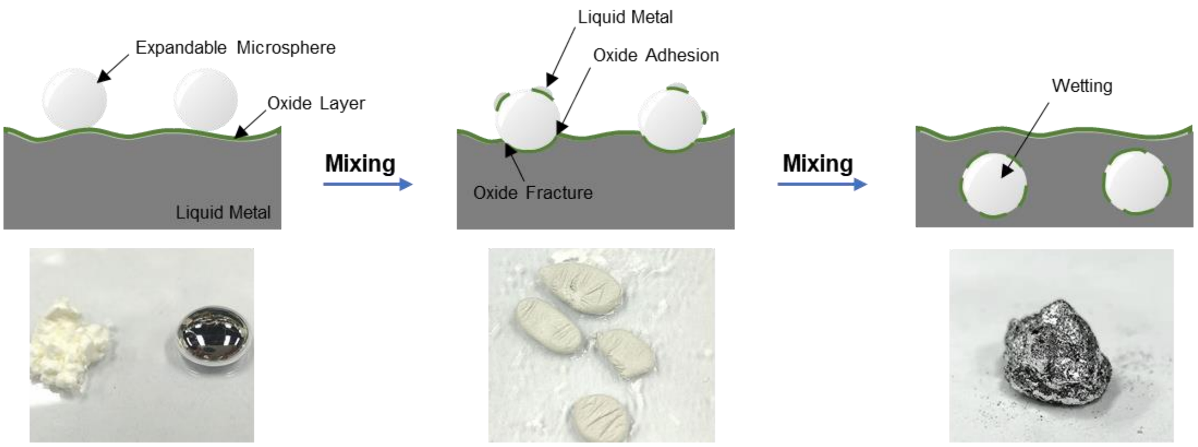

Fig. S3 Illustration of the wetting mechanism of EM/LM compound

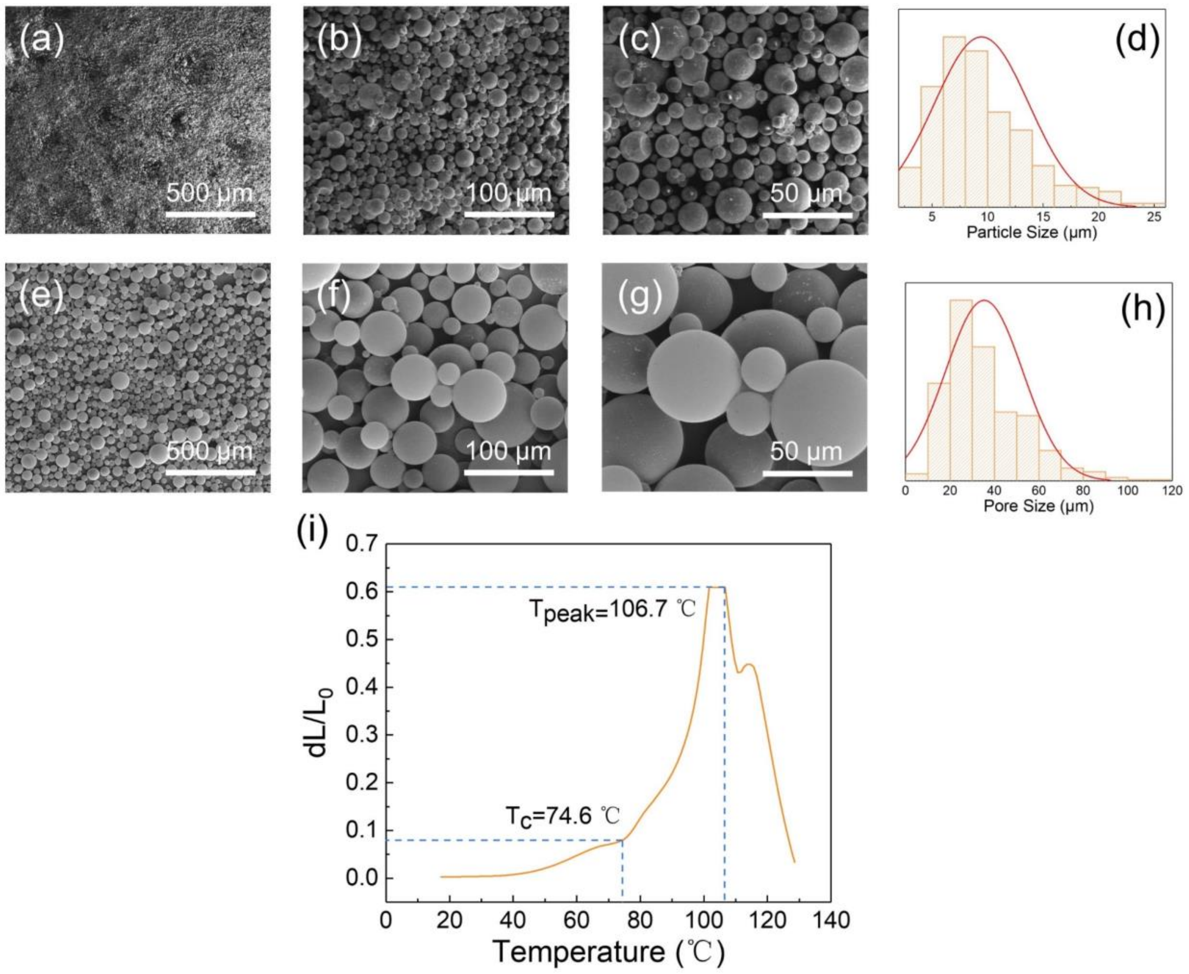

Fig. S4 SEM analysis of size distribution plots of EM before and after expansion. (**a-c**) SEM images with different magnifications of EM before thermal expansion. (**d**) The size distribution statistic shows that the EM particles between 5 and 15 μm. (**e-g**) SEM images at different magnifications of EM after thermal expansion. (**h**) After expansion, the size of EM increased by about 5 times about 10-60 μm. (**i**) The thermal mechanical analyzer curves of EM

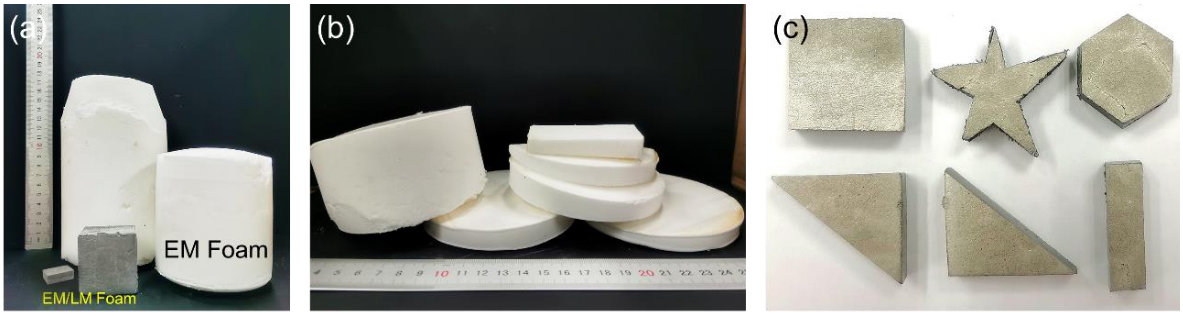

Fig. S5 Optical digital iamges of scaled-up EMm and EM/LMm. (**a**) The mass production and shape control of EMm and EM/LMm. (**b**) General linear cutting can be used for shape and thickness control, indicating machinability of EMm (Movie S1). (**c**) The EM/LMm with different shapes show excellent tailorability

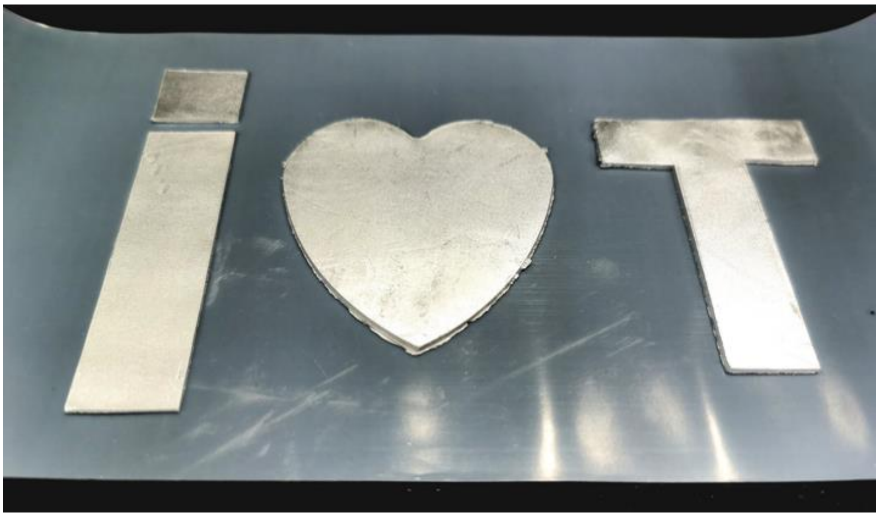

Fig. S6 The reshaping property of EM/LM compounds. It has excellent plasticity and can be moulded into various shapes on demand (i♥T: I Love Technology)

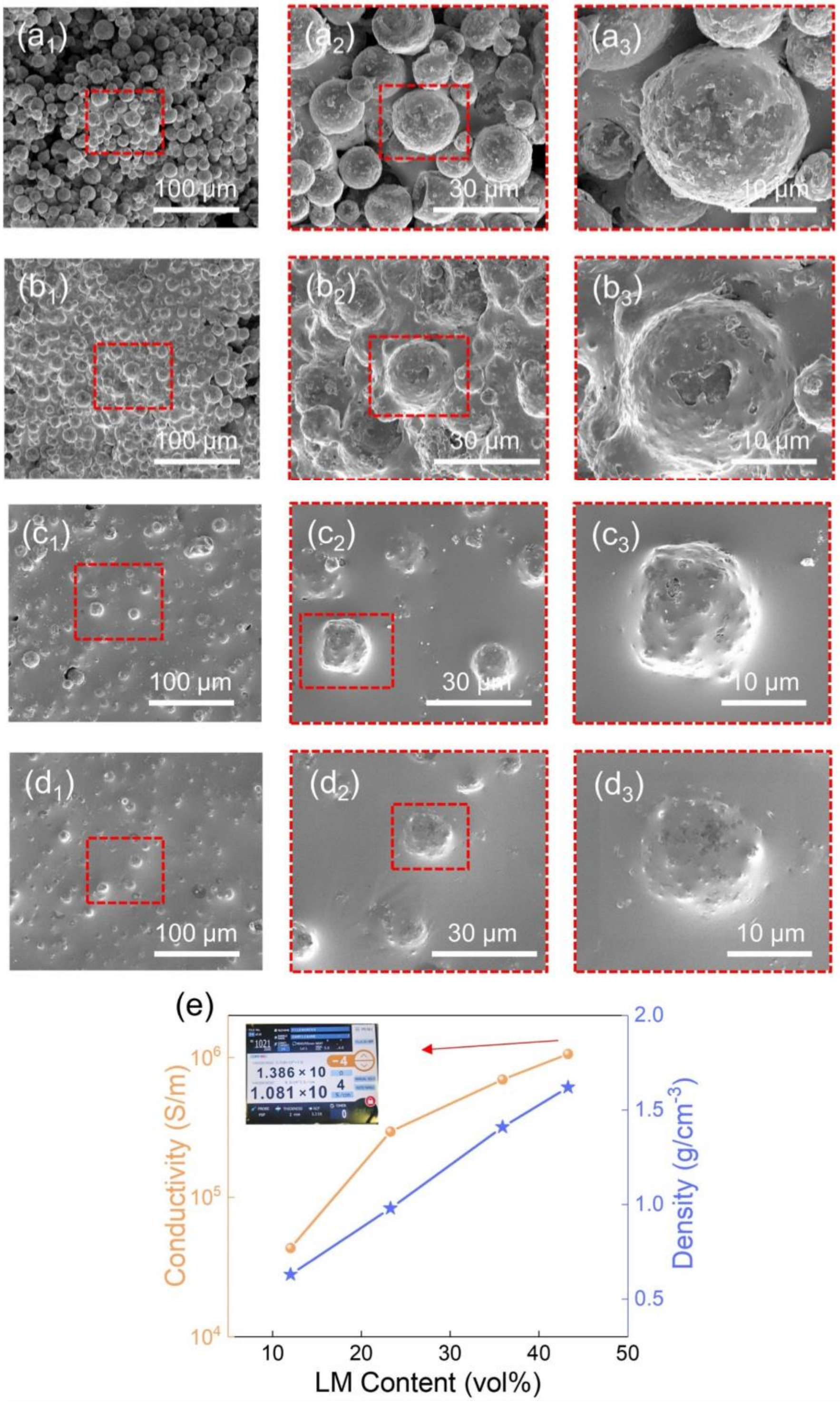

Fig. S7 The SEM images of EM/LM compounds with various weight ratios. (**a**) 3:5, (**b**) 3:15, (**c**) 3:20, (**d**) 3:25. Increasing the LM content, LM is gradually transformed from a surface coated structure to a continuous phase, and then changes in macroscopic physical properties from a solid powder to a viscous paste. (**e**) The density and electrical conductivity of EM/LM compounds with various LM contents

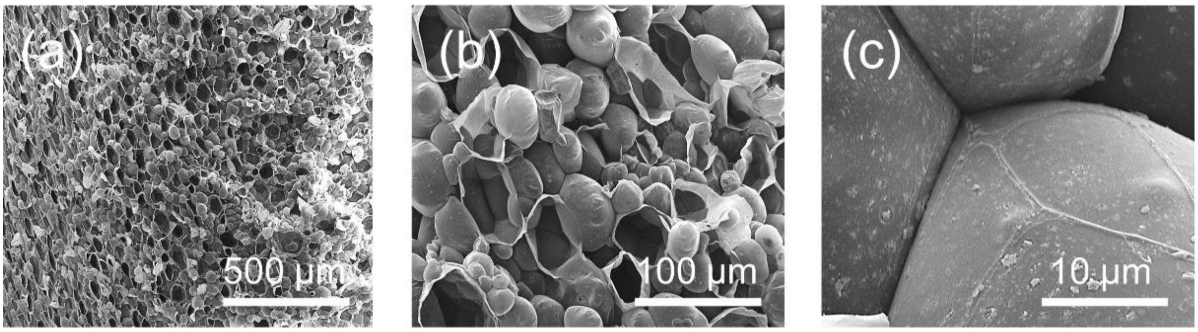

Fig. S8 The formation mechanism from EM powder to EMm. (**a, b**) The SEM cross-sectional images by brittle fracture of EMm exhibits that EM squeezing and sintering each other in a confined space at a certain temperature. (**c**) The traces of mutual sintering remained on the surface

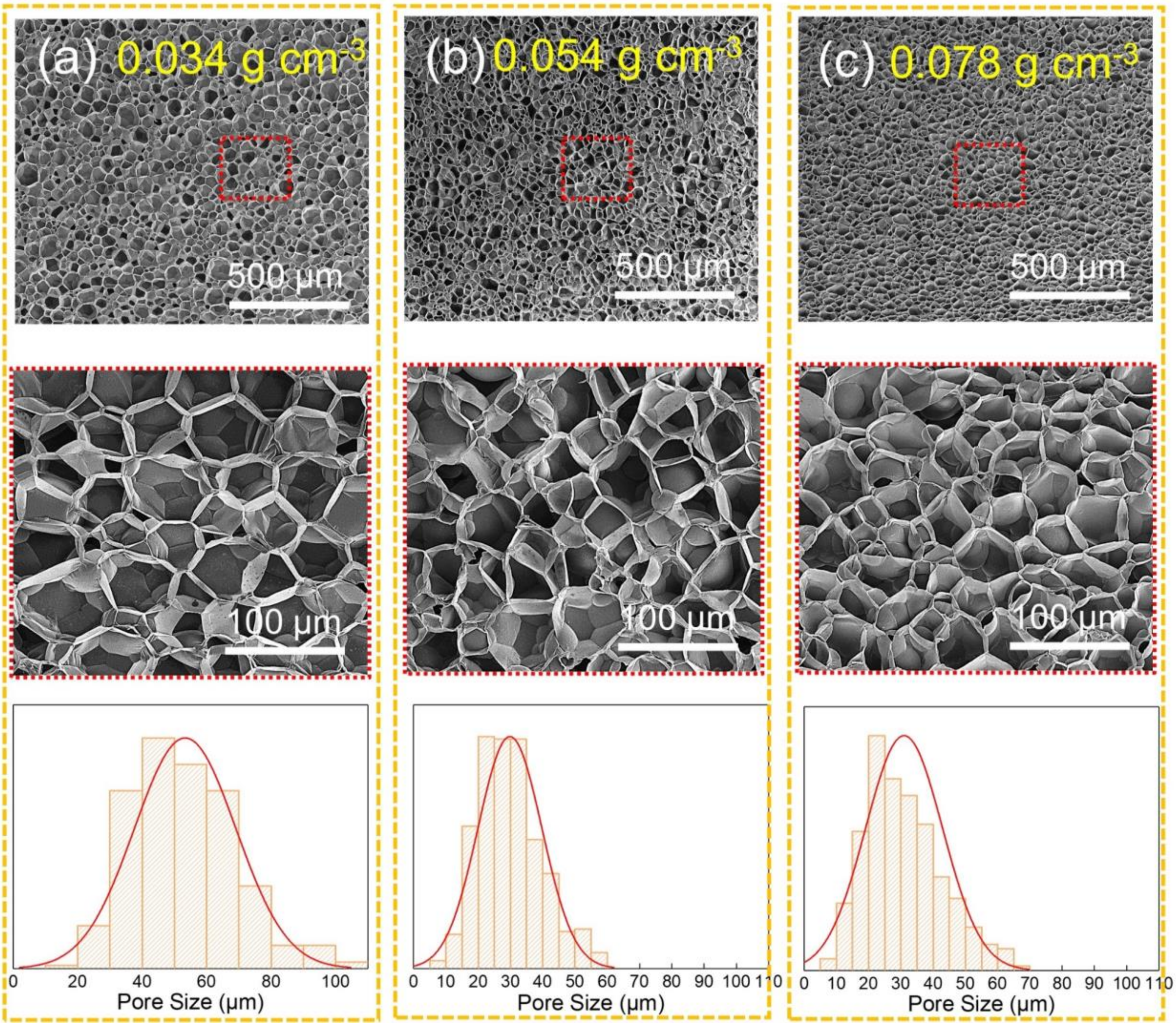

Fig. S9 The cross-sectional SEM images and pore size distribution characteristic of EMm with various densities. The density of EMm was regulated by adding different amounts of EM into the closed mold, (**a**) 0.034 g cm^{-3}, (**b**) 0.054 g cm^{-3}, and (**c**) 0.078 g cm^{-3}. The pore size distribution presents a normal distribution, which decreases gradually with the increase of density. The average pore size decreases from about 50 μm to 20 μm

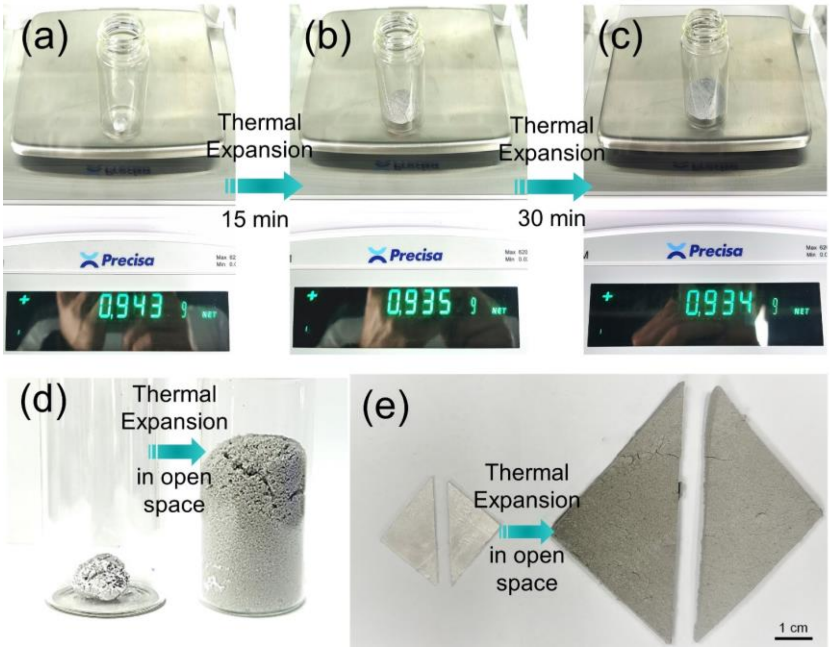

Fig. S10 The weight, volume, and shape changes of EM/LM compounds before and after expansion in open space. (**a-c**) The weight of spherical EM/LM compounds is almost constant during expansion process with only 0.95 wt% loss. (**d**) Visually demonstration of volume changes after thermal expansion of EM/LM compounds in an open glass bottle. (**e**) Visually demonstration of shape retention after thermal expansion of triangular EM/LM compounds in a whole open space. The apparent state of EM/LM compounds changes from plasticine-like state to solid state

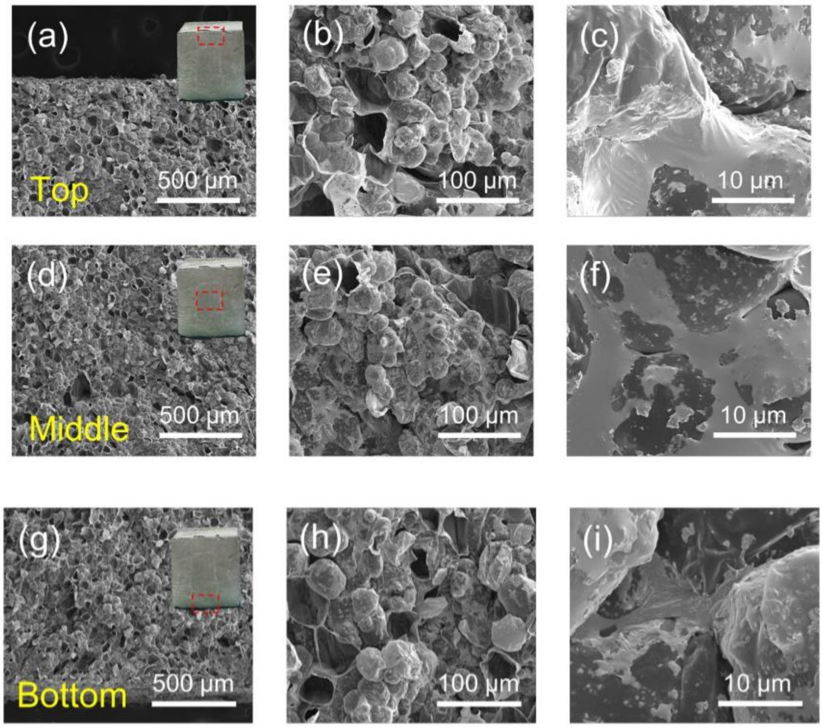

Fig. S11 The distribution analysis of LM in different sections of EM/LMm: (**a-c**) top, (**d-f**) middle, (**g-i**) bottom. The cross-sectional SEM images exhibit that LM presents a uniform distribution in the monoliths, and there is no obvious selective distribution and phase separation due to the expansion process

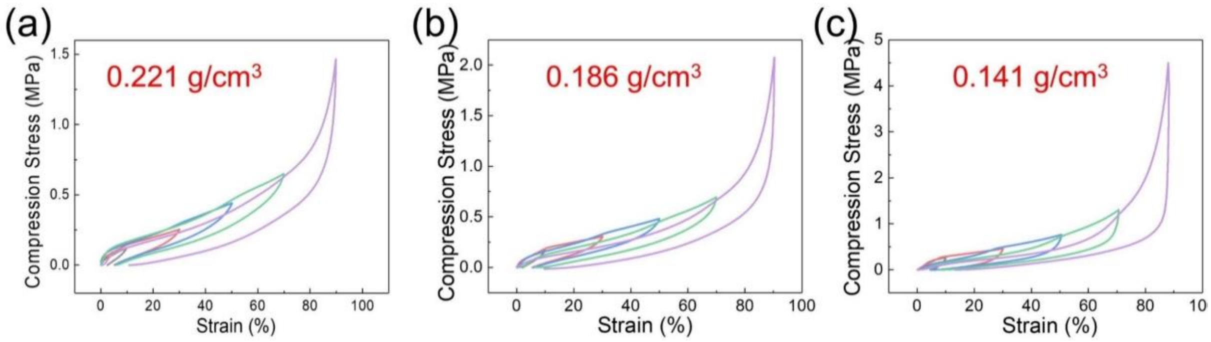

Fig. S12 The compression stress-strain curves of EM/LMm with various density

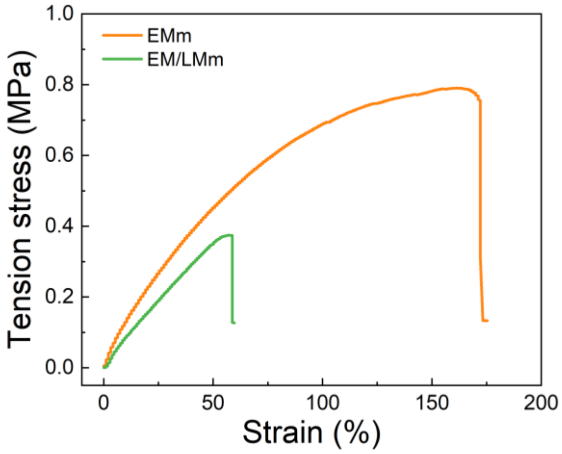

Fig. S13 The tension stress-strain curves of EMm and EM/LMm

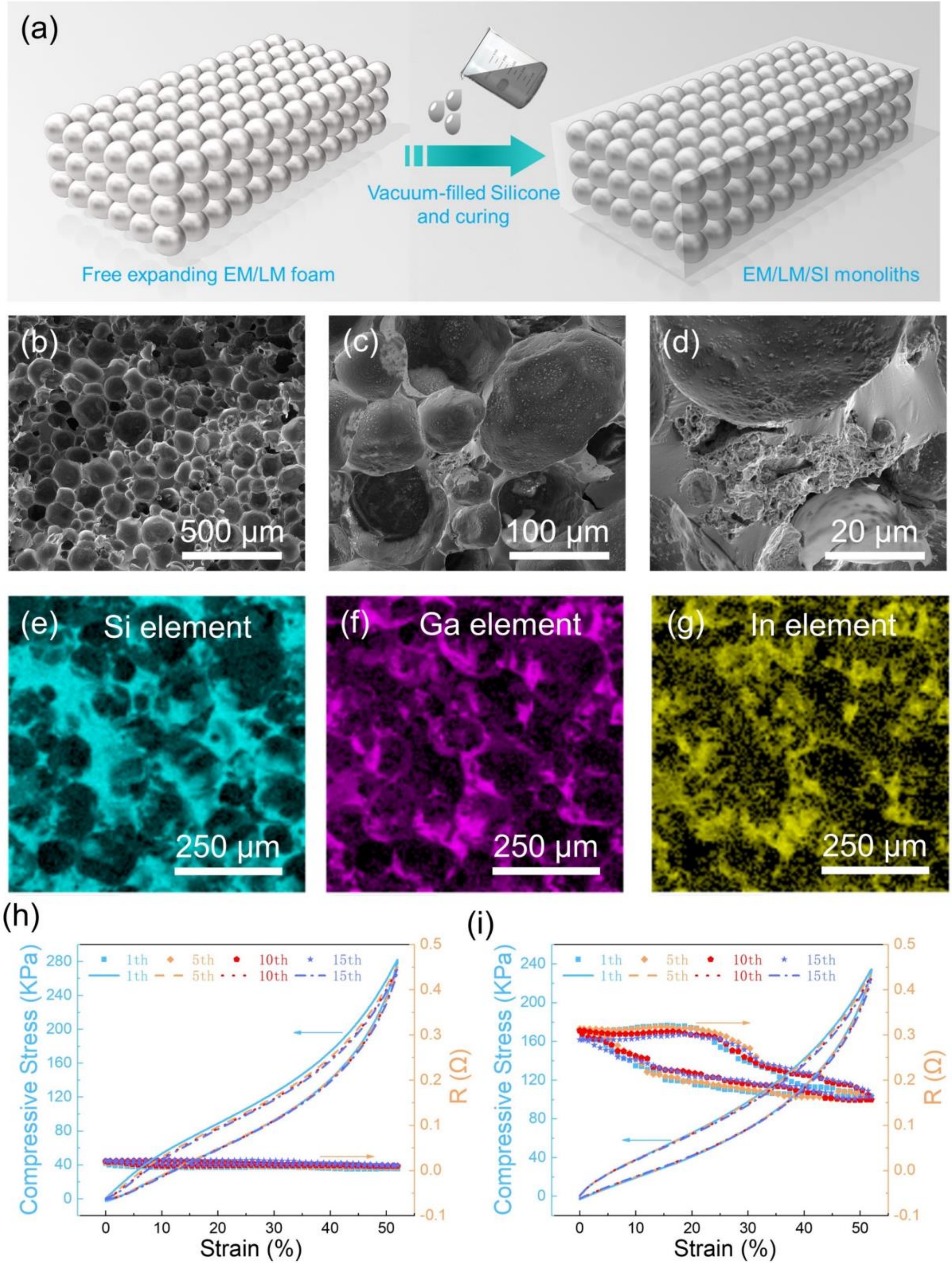

Fig. S14 The cross-sectional SEM, EDS elements mapping and cyclic compression performance characteristic of EM/LM/SI monoliths. (**a**) Illustration of the fabrication process of EM/LM/SI monoliths. (**b-c**) The cross-sectional SEM images show a uniform porous structure, in which the LM is distributed between the EM walls. (**e-g**) The silicone and LM shows uniform distribution in EDS elements mapping images. The mechanical-electronic property of EM/LM/SI monoliths with (**h**) 3.08 vol% of LM and (**i**) 1.85 vol% of LM. With synergistic action of EM and silicone, the EM/LM/SI monoliths shows low relaxation time and weak stress relaxation in cyclic compression test

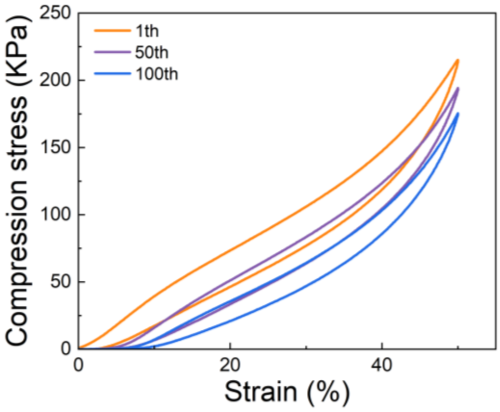

Fig. S15 The compression stress-strain cyclic curves of EM/LM/SI foam under 50% strain

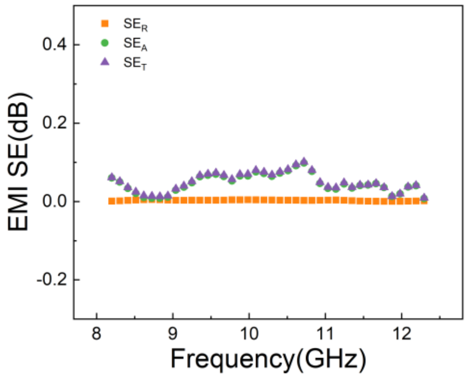

Fig. S16 The EMI SE of EMm in frequency range of 8.2 - 12.4 GHz

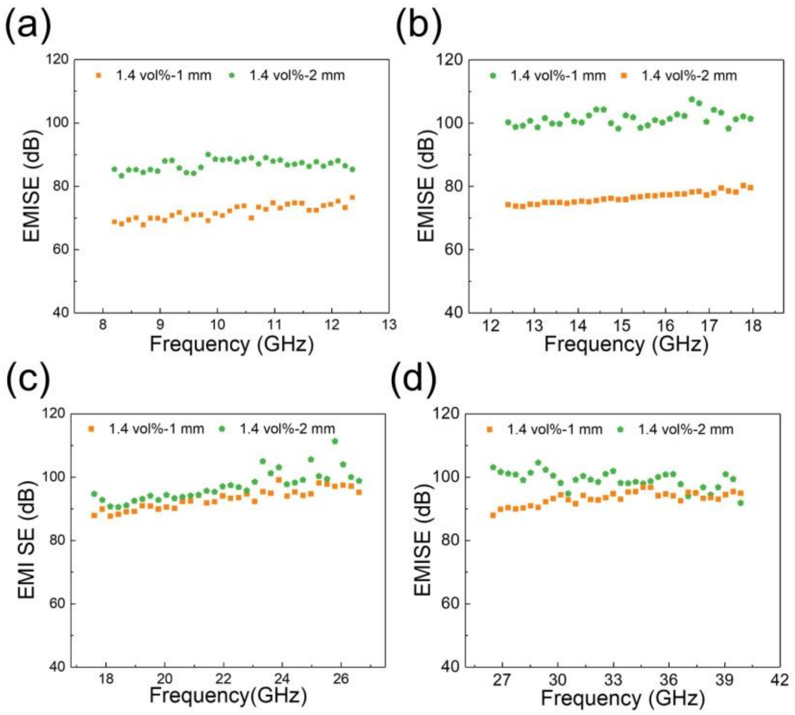

Fig. S17 The EMI SE of EM/LMm with 1.4 vol% of LM at different thicknesses in frequency range of 8.2 - 40 GHz

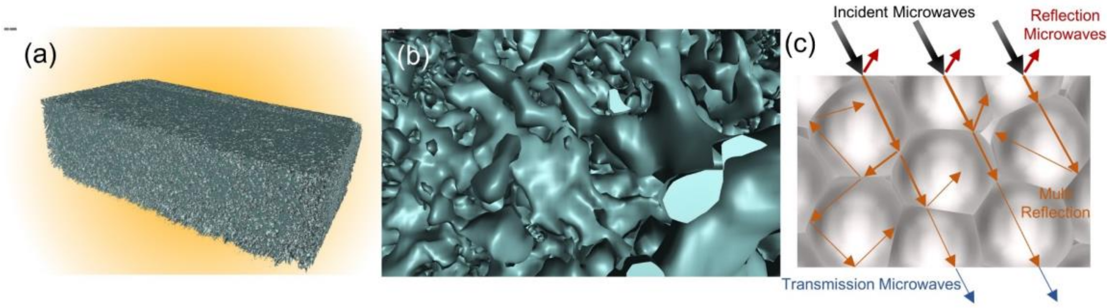

Fig. S18 (a) The reconstructed 3D LM skeleton by Micro-CT. (**b**) The inside microstructure of LM networks of EM/LMm. (**c**) Schematic representation of the shielding mechanism for the EM/LMm

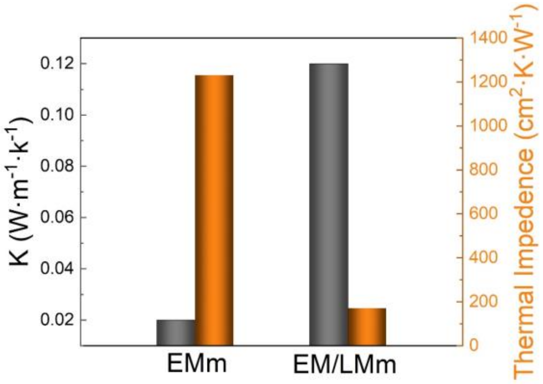

Fig. S19 Thermal conductivity and impedance of EMm and EM/LMm

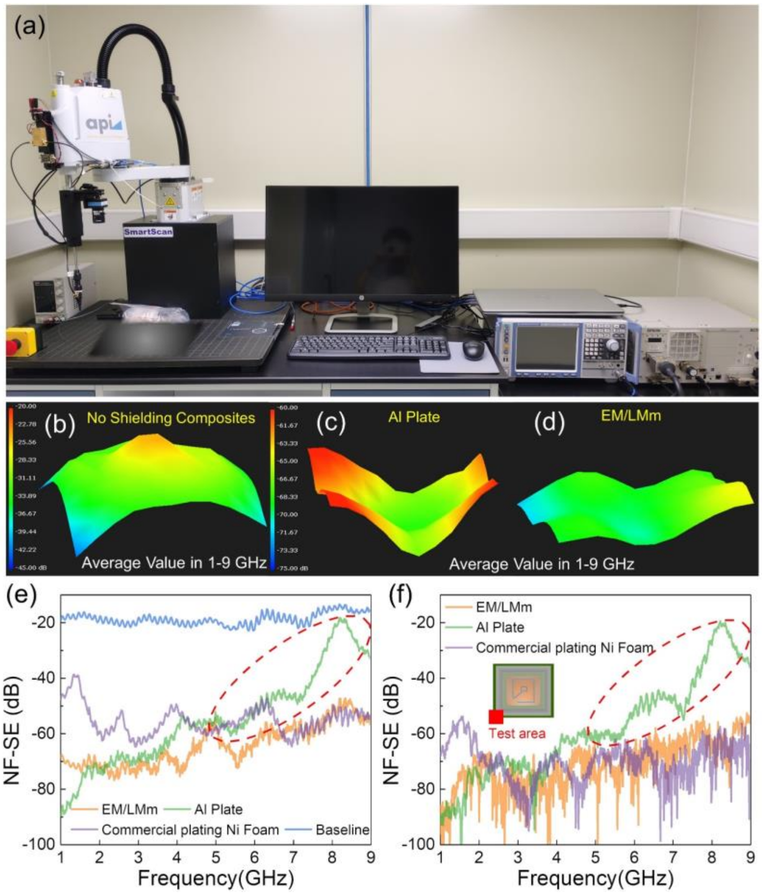

Fig. S20 The NF-SE comparison analysis in different ways (**a**) The test system of NF-SE

performance (**b-d**) The 3D mapping plot of average NF-SE of different shielding materials in 1-9 GHz (**e**) In horizontal test area, the worst NF-SE value at a certain frequency of different shielding materials. The NF-SE of Al plate shows a strong dependence on frequency result from the inferior interfacial contact. The EM/LMm has better NF-SE performance compared to commercial plating Ni foam (**f**) In test area shown in the illustration, the NF-SE of Al plate decreased to some extent in the high frequency area, indicating the leakage of electromagnetic waves signal. The commercial plating Ni foam and EM/LMm have good electromagnetic tightness

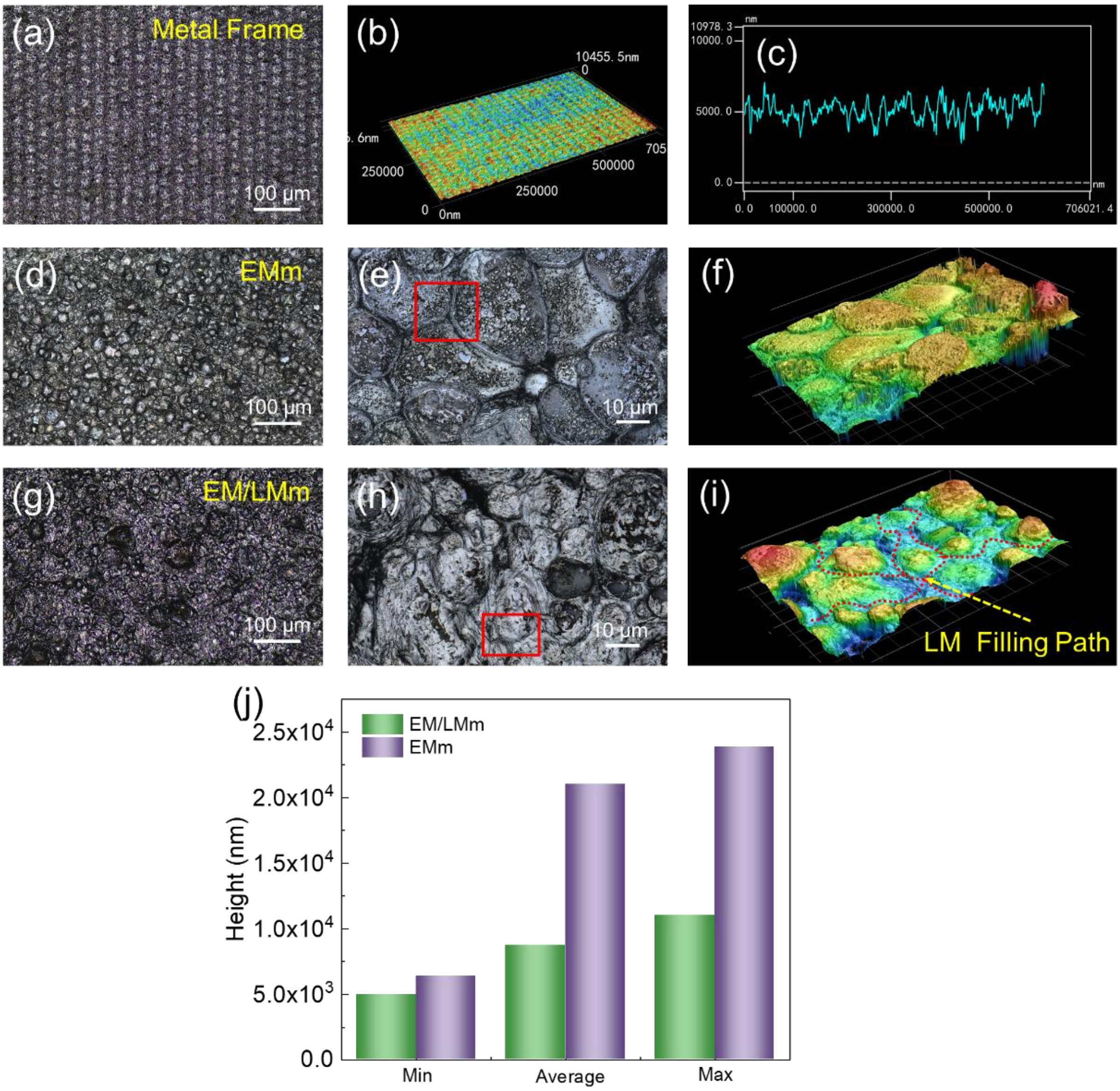

Fig. S21 The laser scanning confocal microscope images for surface roughness analysis. (**a-c**) The optical images, three-dimensional topography and linear roughness of metal frame surface. (**d-f**) The optical images and three-dimensional topography of EMm surface. (**g-i**) The optical images and three-dimensional topography of EM/LMm surface. (**j**) Height statistical comparison of two areas, which are marked with red frame in e and h. The traces of LM on the EM/LMm surface have both the characteristics of migration and stability under pressure

The surface of metal frame matrix is not smooth in micro scale and shows a certain roughness (Fig. S14c), result in incomplete contact between matrix and shielding materials. Compared

with pure EMm (Fig. S14f), the surface of EM/LMm (Fig. S14i) presents a valley-like geology, in which trace amounts of LM exist like rivers in the gaps between EM. By statistically comparing the heights of different materials, it is found that the difference of EM/LMm surface height is obviously weakened, due to the existence of LM between the EM gaps (Fig. S14j).

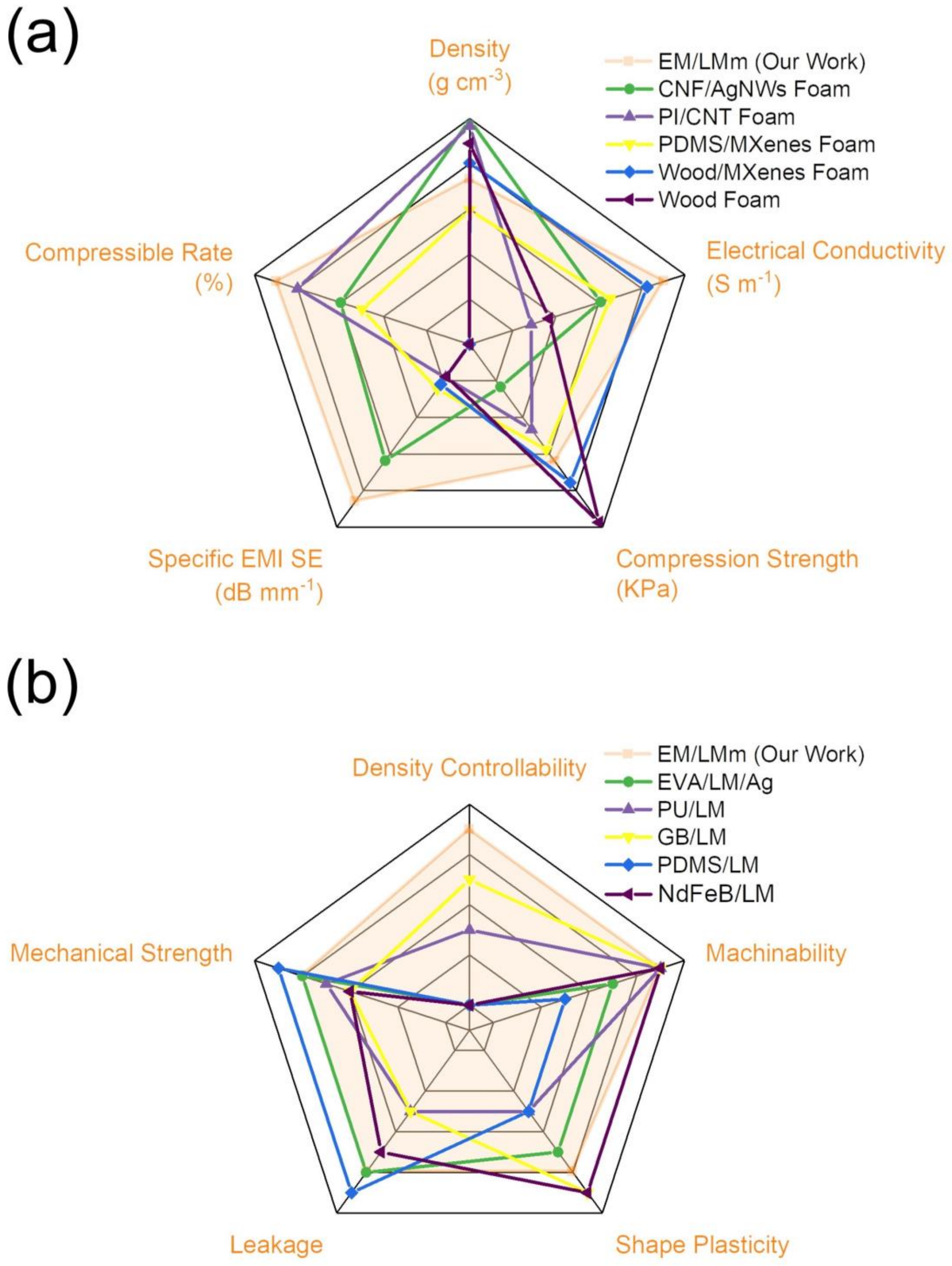

Fig. S22 A radar plot showing a multifaceted comparison of EMm and EM/LMm with (**a**) EMI shielding foam and (**b**) typical LM-based composites, respectively (more details are displayed in Supplementary Table 2, S3)

Table S1 Comparison of comprehensive compression performance of EM/LMm with other compressible elastic polymer-based foams in previous reports.

Materials	Compression Strength (50% strain)	Density (g cm-3)	Reversible Compressive Strain (%)	SCS (N m kg-1)	Refs.
CNF/AgNWs	~3 kPa	0.0017	60	1764	[S1]
Mxene/PI	170 kPa	0.028	80	6071	[S2]
CNFAs	~40 kPa	0.0183	90	2186	[S3]
rGO	~25 kPa	0.00466	80	5365	[S4]
Melamine/GO	~20 kPa	0.12	70	167	[S5]
Aramid /MXene	~30 kPa (30%)	0.025	80	1200	[S6]
Wood/MXene	~40 kPa	~0.15	60	267	[S7]
RGO/LDC	~3 kPa	~0.012	70	250	[S8]
SiC	~3 kPa	~0.0065	60	462	[S9]
RGO	~7 kPa	~0.0078	70	897	[S10]
EMm	520 kPa	0.05	95	10400	Our work
EM/LMm	610 kPa	0.225	90	2711	Our work

Table S2 Multifaceted comparison of EM/LMm with EMI shielding foam (corresponding to Fig. S15a).

Materials	Density (g cm-3)	Electrical Conductivity (S m-1)	Compression Strength (50% strain) (kPa)	Specific EMI SE (dB mm-1)	Compressive Recovery Rate (%)	Refs.
CNF/AgNWS	0.0062	416	10	70	60	[S1]
PI/CNT	0.032	17.1	100	20	80	[S11]
PDMS/MXene	0.405	644	300	26.9	50	[S12]
Carbide Wood/MXene	0.197	3520	1800	24	No	[S13]
Carbide Wood	0.11	39	15460	19.3	No	[S14]
EM/LMm	0.225	5541	610	98.7	~90	Our Work

Table S3 Multifaceted comparison of EMm and EM/LMm with typical LM-based composites (corresponding to Fig. S15b).

Materials	Density Controllability	Machinability	Shape Plasticity	Leakage Proof	Mechanical Strength	Refs.
EVA/LM/Ag	(bulk composite)<br>2	(solution blending)<br>7	(ink)<br>7	(EVA package)<br>8	(stretchable)<br>8	[S15]
PU/LM	(foam or composite)<br>5	(dip coating)<br>9	(foam)<br>5	(no package)<br>5	(compressible)<br>7	[S16]
GB/LM	(0.5 g/cm3)<br>7	(mechanical mixing)<br>9	(paste)<br>9	(PU film package)<br>5	(plasticity)<br>6	[S17]
PDMS/LM	(bulk composite)<br>2	(sacrifice template)<br>5	(composite)<br>5	(PDMS package)<br>9	(stretchable)<br>9	[S18]
NdFeB/LM	(bulk composite)<br>2	(mechanical mixing)<br>9	(plasticine)<br>9	(no package)<br>7	(plasticity)<br>6	[S19]
EM/LMm	(0.104 g/cm3)<br>9	(mechanical mixing)<br>9	(paste or foam)<br>8	(low content and EM package)<br>8	(compressible)<br>8	Our Work

NOTES: The attributes of composites are rated on a ten (10) point scale.

Supplementary References

[S1] Z. Zeng, T. Wu, D. Han, Q. Ren, G. Siqueira et al., Ultralight, flexible, and biomimetic nanocellulose/silver nanowire aerogels for electromagnetic interference shielding. ACS Nano **14**(3), 2927-2938 (2020). https://doi.org/10.1021/acsnano.9b07452

[S2] J. Liu, H.B. Zhang, X. Xie, R. Yang, Z. Liu et al., Multifunctional, superelastic, and

lightweight MXene/polyimide aerogels. Small **14**(45), e1802479 (2018). https://doi.org/10.1002/smll.201802479

[S3] C. Li, Y.W. Ding, B.C Hu, Z.Y. Wu, H.L. Gao et al., Temperature-invariant superelastic and fatigue resistant carbon nanofiber aerogels. Adv. Mater. **32**(2), e1904331 (2020). https://doi.org/10.1002/adma.201904331

[S4] S.J. Yeo, M.J. Oh, H.M. Jun, M. Lee, J.G. Bae et al., A plesiohedral cellular network of graphene bubbles for ultralight, strong, and superelastic materials. Adv. Mater. **30**(45), e1802997 (2018). https://doi.org/10.1002/adma.201802997

[S5] J. Xi, Y. Liu, Y. Wu, J. Hu, W. Gao et al., Multifunctional bicontinuous composite foams with ultralow percolation thresholds. ACS Appl. Mater. Interfaces **10**(24), 20806-20815 (2018). https://doi.org/10.1021/acsami.8b06017

[S6] L. Wang, M. Zhang, B. Yang, J. Tan, X. Ding, Highly compressible, thermally stable, light-weight, and robust aramid nanofibers/Ti_3AlC_2 MXene composite aerogel for sensitive pressure sensor. ACS Nano **14**(8), 10633-10647 (2020). https://doi.org/10.1021/acsnano.0c04888

[S7] X. Jia, B. Shen, L. Zhang, W. Zheng, Construction of shape-memory carbon foam composites for adjustable EMI shielding under self-fixable mechanical deformation. Chem. Eng. J. **405**, 126927 (2021). https://doi.org/10.1016/j.cej.2020.126927

[S8] Z. Zeng, C. Wang, Y. Zhang, P. Wang, S.I.S. Shahabadi et al., Ultralight and highly elastic graphene/lignin-derived carbon nanocomposite aerogels with ultrahigh electromagnetic interference shielding performance. ACS Appl. Mater. Interfaces **10**(9), 8205-8213 (2018). https://doi.org/10.1021/acsami.7b19427

[S9] L. Su, H. Wang, M. Niu, S. Dai, Z. Cai et al., Anisotropic and hierarchical SiC@SiO_2 nanowire aerogel with exceptional stiffness and stability for thermal superinsulation. Sci. Adv. **6**(26), eaay6689 (2020). https://doi.org/10.1126/sciadv.aay6689

[S10] X. Zhao, W. Yao, W. Gao, H. Chen, C. Gao, Wet-spun superelastic graphene aerogel millispheres with group effect. Adv. Mater. **29**(35), 1701482 (2017). https://doi.org/10.1002/adma.201701482

[S11] Y.Y. Wang, Z.H. Zhou, C.G. Zhou, W.J. Sun, J.F. Gao et al., Lightweight and robust carbon nanotube/polyimide foam for efficient and heat-resistant electromagnetic interference shielding and microwave absorption. ACS Appl. Mater. Interfaces **12**(7), 8704-8712 (2020). https://doi.org/10.1021/acsami.9b21048

[S12] X. Wu, B. Han, H.B. Zhang, X. Xie, T. Tu et al., Compressible, durable and conductive polydimethylsiloxane-coated MXene foams for high-performance electromagnetic interference shielding. Chem. Eng. J. **381**, 122622 (2020). https://doi.org/10.1016/j.cej.2019.122622

[S13] C. Liang, H. Qiu, P. Song, X. Shi, J. Kong et al., Ultra-light MXene aerogel/wood-derived porous carbon composites with wall-like "mortar/brick" structures for electromagnetic interference shielding. Sci. Bull. **65**(8), 616-622 (2020). https://doi.org/10.1016/j.scib.2020.02.009

[S14] W. Gan, C. Chen, M. Giroux, G. Zhong, M.M. Goyal et al., Conductive wood for high-performance structural electromagnetic interference shielding. Chem. Mater. **32**(12), 5280-5289 (2020). https://doi.org/10.1021/acs.chemmater.0c01507

[S15] J. Wang, G. Cai, S. Li, D. Gao, J. Xiong et al., Printable superelastic conductors with

extreme stretchability and robust cycling endurance enabled by liquid-metal particles. Adv. Mater. **30**(16), e1706157 (2018). https://doi.org/10.1002/adma.201706157

[S16] Y. Huang, B. Yu, L. Zhang, N. Ning, M. Tian, Highly stretchable conductor by self-assembling and mechanical sintering of a 2D liquid metal on a 3D polydopamine-modified polyurethane sponge. ACS Appl. Mater. Interfaces **11**(51), 48321-48330 (2019). https://doi.org/10.1021/acsami.9b15776

[S17] B. Yuan, C. Zhao, X. Sun, J. Liu, Lightweight liquid metal entity. Adv. Funct. Mater. **30**(14), 1910709 (2020). https://doi.org/10.1002/adfm.201910709

[S18] B. Yao, W. Hong, T. Chen, Z. Han, X. Xu et al., Highly stretchable polymer composite with strain-enhanced electromagnetic interference shielding effectiveness. Adv. Mater. **32**(14), e1907499 (2020). https://doi.org/10.1002/adma.201907499

[S19] L. Cao, D. Yu, Z. Xia, H. Wan, C. Liu et al., Ferromagnetic liquid metal putty-like material with transformed shape and reconfigurable polarity. Adv. Mater. **32**(17), e2000827 (2020). https://doi.org/10.1002/adma.202000827
